# Filtering Organized 3D Point Clouds for Bin Picking Applications

**DOI:** 10.3390/app14030961

**Published:** 2024

**Authors:** Marek Franaszek, Prem Rachakonda, Kamel S. Saidi

**Affiliations:** National Institute of Standards and Technology, Gaithersburg, MD 20899, USA;

**Keywords:** statistical outlier removal, filtering 3D point cloud, bin picking, segmentation

## Abstract

In robotic bin-picking applications, autonomous robot action is guided by a perception system integrated with the robot. Unfortunately, many perception systems output data contaminated by spurious points that have no correspondence to the real physical objects. Such spurious points in 3D data are the outliers that may spoil obstacle avoidance planning executed by the robot controller and impede the segmentation of individual parts in the bin. Thus, they need to be removed. Many outlier removal procedures have been proposed that work very well on unorganized 3D point clouds acquired for different, mostly outdoor, scenarios, but these usually do not transfer well to the manufacturing domain. This paper presents a new filtering technique specifically designed to deal with the organized 3D point cloud acquired from a cluttered scene, which is typical for a bin-picking task. The new procedure was tested on six different datasets (bins filled with different parts) and its performance was compared with the generic statistical outlier removal procedure. The new method outperforms the general procedure in terms of filtering efficacy, especially on datasets heavily contaminated by numerous outliers.

## 1. Introduction

**Figure 1.**
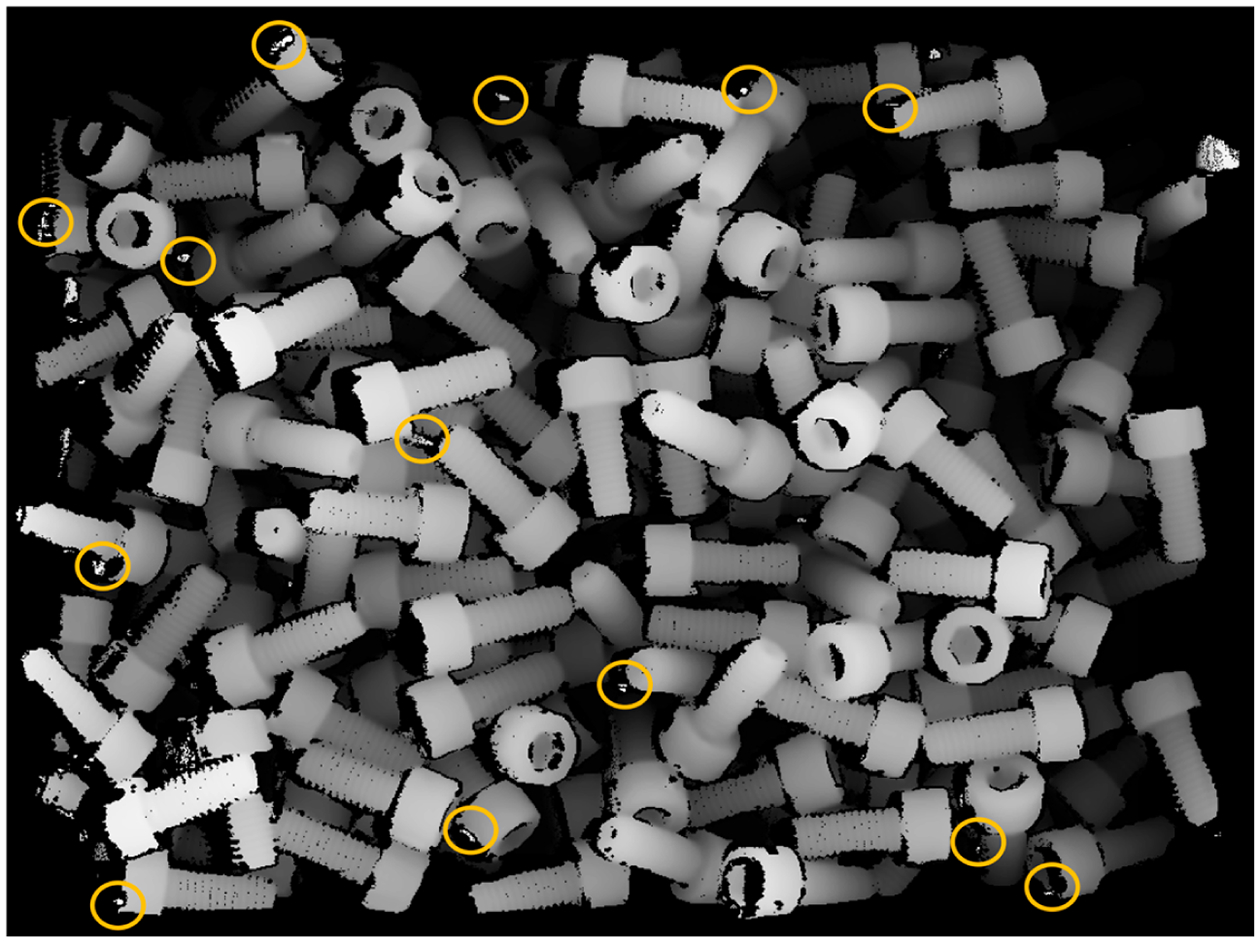
Example of depth map for bin-picking. Marked by colored ellipse are some examples of outliers (bright pixels indicating points with unusually small depth, i.e., high z).

Robotic bin-picking is a common task in manufacturing [1]. Its objective is to pick a single part from an unstructured pile in a box, as shown in Figure 1, and make the part available for the next step of the automated process [2].

**Figure 2.**
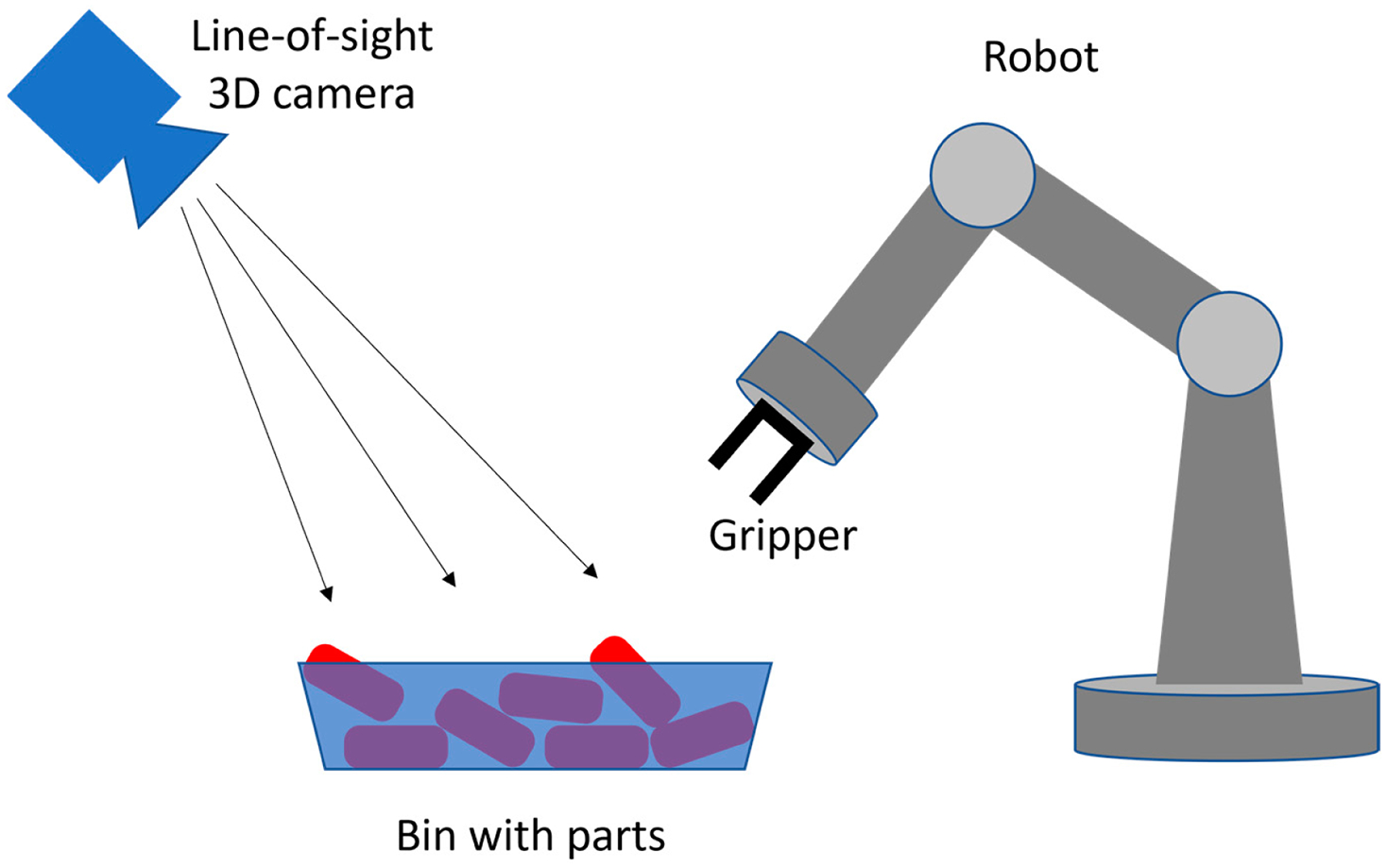
Schematic drawing of a typical setup for robotic bin-picking applications.

High levels of autonomy and easily reconfigurable work cells are needed to achieve a smooth flow of the production lines. This is often achieved using 3D machine vision systems that are integrated with the robots. A schematic configuration of a typical setup, with a 3D camera and robot in fixed locations in a world coordinate frame, is shown in Figure 2. Vision systems acquire and process 3D data to provide the robot controller with spatial awareness for path planning. Planning includes the selection of a single part from a pile of parts and the design of a safe path from the starting gripper pose to the grasp pose for the selected part. Poor quality of the acquired data and erroneous results provided by a vision system may severely degrade the performance of a robot. Spurious, unfiltered 3D points provided by a vision system can be interpreted by the obstacle avoidance function as a real object and may degrade the robot’s path planning algorithm. Similarly, spurious 3D points will cause an incorrectly determined six-degree-of-freedom (6DOF) pose of the selected single part in a bin, which will likely lead to an unsuccessful attempt to grip this part by a robot [3].

The complexity of the bin-picking process varies greatly from application to application, which affects the appropriate type of machine vision hardware and processing algorithm for a particular scenario [4]. For example, if a bin contains quasi-2D objects, a single camera and an algorithm based on well-known 2D image processing techniques may be sufficient [5]. Similarly, if parts in a bin are lightweight and a vacuum gripper can be used, less accurate segmentation of a single part and its derived 6DOF pose may still be acceptable. This may not be the case for parts that cannot be considered as quasi-2D (i.e., parts where all three dimensions are comparable) as they require more precise grasping by a gripper [6]. Another factor that impacts the complexity of bin-picking is the finish of the part’s surface. For example, shiny objects are known to yield poor-quality data, and these types of objects are commonly picked from assembly lines in industrial applications [7].

**Figure 3.**
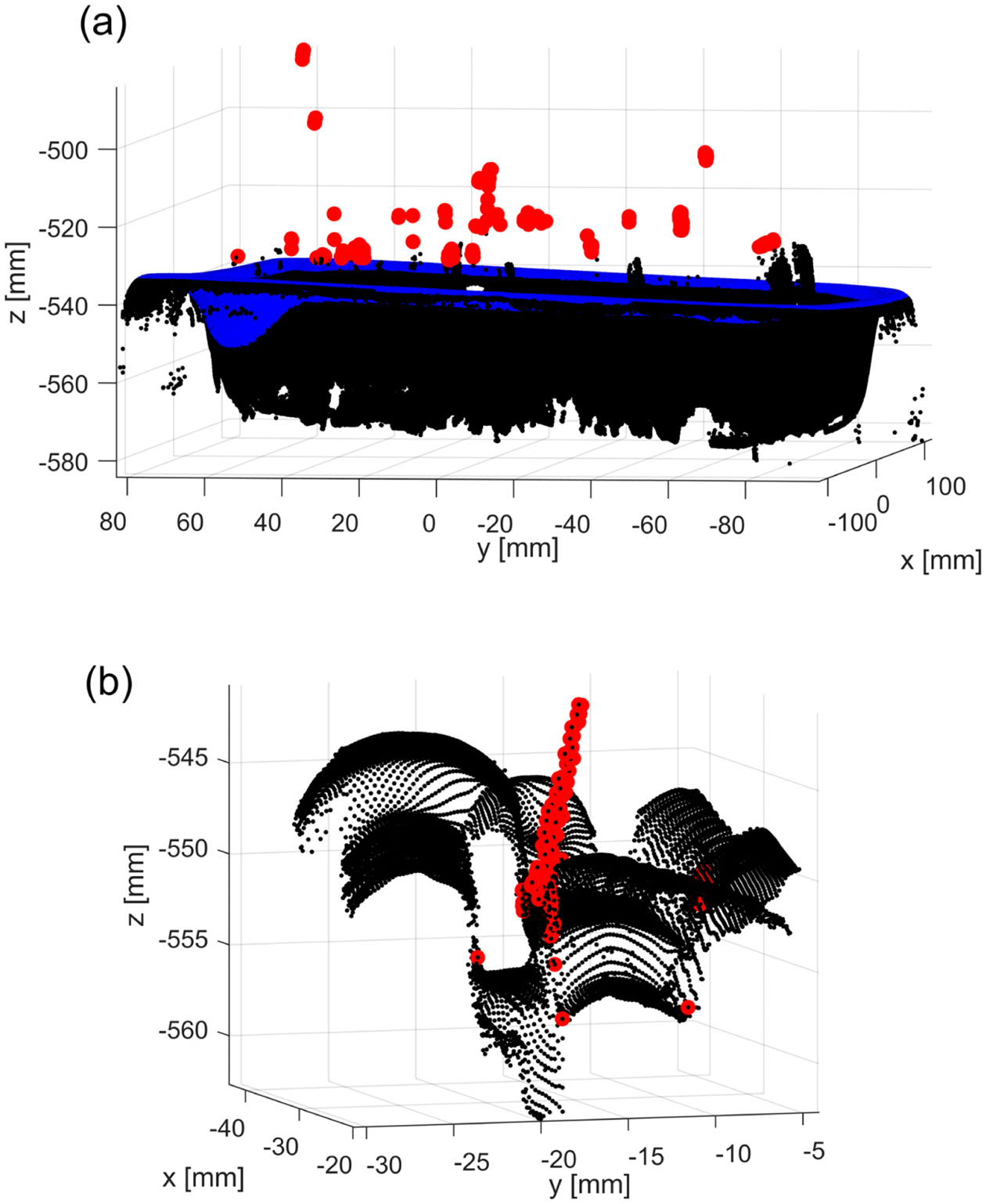
Example of a 3D point cloud of a bin filled with parts: (**a**) red dots are outliers above the top edge of a bin (marked in blue); (**b**) zoomed-in subregion after outliers in (**a**) were removed—red dots show unremoved outliers.

Thus, enhancing the quality of the data acquired and processed by a vision system is important. Different types of machine vision systems have been integrated with robotic arms for different bin-picking scenarios [8–15]. The choice of a particular system determines how the acquired data needs to be addressed by the post-processing algorithms. Systems based on laser scanning or structured light (commonly used on automated production lines) are known to output 3D point clouds, which are contaminated by spurious points floating in small regions with no correspondence to the real, physical objects. Such points are outliers that need to be removed from a dataset before further processing. Portions of these outliers can be easily identified and removed by simple thresholding (depth or z coordinate), as shown in Figure 3a. However, the remaining parts of the 3D point cloud may still contain unfiltered outliers, which may spoil the segmentation of individual parts, as shown in Figure 3b.

This paper presents a new method of filtering these difficult-to-identify outliers from the organized 3D point cloud acquired for bin-picking. The method identifies groups of 3D points with z components separated from the surrounding points by a distance Δd. For the organized 3D points clouds acquired with the line-of-sight system, such groups contain points that float in space disconnected from other data points, obtained from the surfaces of real, physical objects.

The organized 3D point cloud is a set of points $P_{n}=\left(x_{n},y_{n},z_{n}\right),n=1,\ldots ,N$, such that $n=row\;\times N_{col}+\;col;\;row\;=0,\ldots ,N_{row}-1,col=0,\ldots ,N_{col}-1.$ Thus, $N=N_{\text{row}\;}N_{\text{col}}$ and each Cartesian coordinate of a set point can be represented as a matrix of size $N_{\text{row}}\times N_{\text{col}}$, for example, Z=z(row, col). Most 3D machine vision systems have an option to output the data in an organized point cloud format (unorganized point clouds still provide a set of 3D points $P_{n}$, but their components do not form a regular 2D matrix that corresponds to the pixels on the sensor plane). The organized 3D point cloud format enables the use of 2D image processing techniques (many of which are based on the concept of pixel connectivity), while voxelization of the unorganized 3D point cloud is a more complex and time-consuming process.

The proposed filtering method was tested on six datasets, obtained by scanning six bins filled with different parts with a line-of-sight vision system. Recorded 3D point clouds were processed with the generic statistical outlier removal (SOR) algorithm and the proposed procedure. SOR could filter only a small portion of outliers: a majority of the correctly identified outliers relevant for bin-picking were identified by the new procedure.

## 2. Related Work on Filtering 3D Point Clouds

The quality of 3D point clouds depends greatly on the sensor hardware, software settings, and the choice of processing algorithms. For example, the accuracy of the camera calibration has an important effect on object localization [16]. Most commercial vision systems offer the user a set of adjustable parameters (such as lens aperture, exposure time, and sensor gain) and a list of built-in filtering algorithms. In this paper, we focus on the post-processing algorithms, such as those reviewed in [17]. These algorithms try to address two deficiencies in the acquired 3D point clouds: removing outliers and reducing noise. The distinction between these is subject to interpretation, but for point clouds obtained from objects for which computer-aided design (CAD) models are known, the distinction can be based on the distance between a datapoint and the closest vertex, or face, of the *correctly* oriented CAD model [18]. For such cases, we consider a 3D point as noisy when its distance to the CAD model is smaller than 3×Err, where Err is the residual error from fitting the CAD model to the 3D point cloud using, for example, the iterative closest point (ICP) or any other minimization technique. For such defined noisy points, the outlier may be defined as a point with a distance to the CAD model larger than 3×Err. This paper focuses on detecting and filtering these outliers.

The most commonly used filtering techniques belong to the family of statistical outlier removal (SOR) algorithms [19]. They share a similar strategy: neighboring points around each datapoint are selected, and a statistical parameter, such as the mean or standard deviation of the distances between the selected point and its nearest neighbors, is calculated. After the statistical parameter is calculated for each datapoint, a thresholding technique is applied to reject all datapoints that have their parameters above a certain threshold. Different implementations of SOR use different approaches to build a neighborhood or use different statistical parameters. For example, the $L_{0}$ norm was used in [20], while box plots and quartiles were used to throw out outliers in scans of a building in [21]. The distance of a point to the locally fitted plane and the robust Mahalanobis distance were used in [22]. The voxelization of 3D point clouds was used to build a neighborhood in [23]. Multiple scans of the same object acquired from different viewpoints were used to get a weighted average from distances between a checked point and intersecting points on nearby local surfaces [24]. Fast cluster SOR was designed specifically to speed up filtering in large datasets acquired by a sensor mounted on an unmanned ground vehicle (UGV) [25]. A median filter applied to a depth image acquired by a lidar in rainy or snowy weather conditions was demonstrated in [26]. In [27], outlier removal was performed in two steps: first, isolated clusters were removed based on local density and then, using local projection techniques, the remaining non-isolated outliers were removed.

Another class of filtering techniques is available when a red-green-blue depth (RGB-D) camera is used. Such sensors provide not only the organized 3D point cloud but also the depth and RGB images. In [28], the filtering of 3D point clouds was performed by converting the RGB image to a hue saturation value (HSV) image and segmenting the grayscale V image using the Otsu method [29]: the calculated binary mask was then used to extract filtered 3D points.

Another approach to filtering 3D point clouds is derived from machine or deep learning (ML/DL) techniques. The goal of these techniques is to correctly classify inliers and outliers from the acquired data. In [30], the 3D data were first preprocessed with an SOR-type procedure and then points marked as outliers were reclassified using random forest (RF) and support vector machine (SVM) techniques. Isolation forest (IF) and elliptic envelope (EE) methods were used to classify outliers in synthetic data and two scans of a building [31]. Fully connected network (FNN) and spatial transformer network (STN) methods were used in [32] to first remove outliers and then to perform denoising. A standard RF classifier was used in two approaches, non-semantic and semantic (i.e., with a classifier trained separately per each semantic class), and applied to large datasets of outdoor scenes [33]. Removal of outliers in a pair of point clouds of the same scene acquired from two viewpoints was demonstrated in [34].

While most of the reviewed techniques present very impressive results, they are demonstrated on cases that are outside of the scenarios relevant in manufacturing for bin-picking. Many techniques were tested on outdoor scans or datasets containing only one object [35]. Point clouds acquired for bin-picking have completely different characteristics: the most noticeable is a presence of many instances of the same part, which are touching and occluding each other. This makes the removal of outliers very challenging. On the other hand, for bin-picking applications, not all outliers are important and need to be filtered, as only parts that are on the top of a pile are relevant for picking by the gripper. In this paper, we present a novel approach that is designed to address specific characteristics of outliers (spurious points) commonly found in 3D data acquired for bin-picking. This new fine-tuned algorithm outperforms existing general-purpose SOR procedures in terms of filtering efficacy, gauged by a number of the removed outliers.

## 3. New Filtering Method

**Figure 4.**
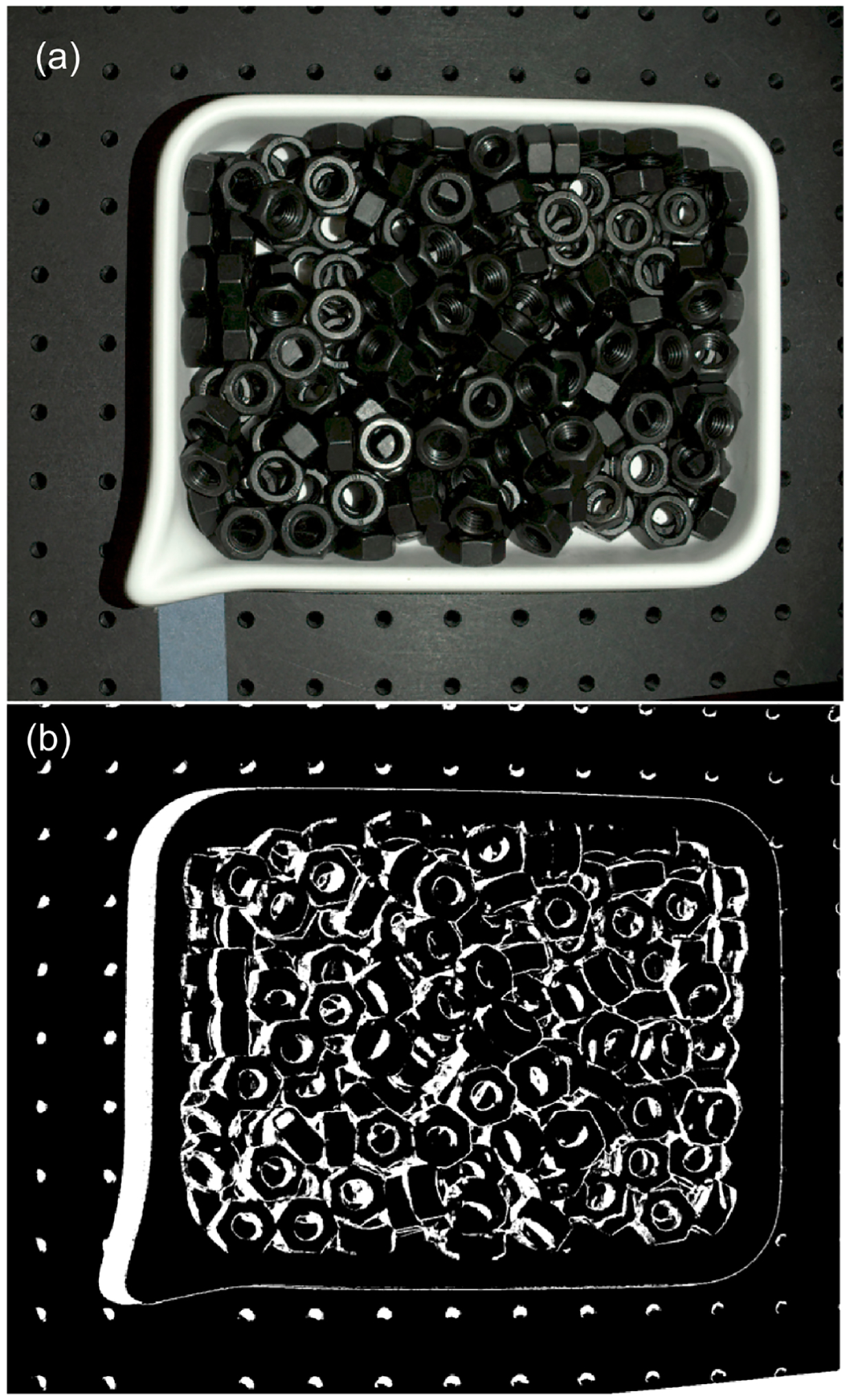
Example of different formats of data output: (**a**) RGB image; (**b**) binary map, white pixels correspond to NaN pixels.

As mentioned in the introduction, for organized point clouds, each Cartesian coordinate of a dataset can be represented as a matrix of size $N_{\text{row}}\times N_{col}$, for example, Z=z(row,col). In the underlying RGB image, each of its pixels has three assigned valid numerical values (for red, green, and blue components). For the organized 3D point cloud, the data structure is more complicated as some pixels may not have valid entries, as shown in Figure 4. This happens when the calculation of the depth value corresponding to a given pixel fails. The reason for such failures depends on the underlying sensor technology. For example, for time-of-flight sensors, it could be the oversaturation or undersaturation of the receiver. For triangulation-based systems, it could be a failure to find pixel correspondence due to object occlusions and shadows.

The locations of such pixels in an organized point cloud are usually marked with NaN (Not a Number) or filled with (0, 0, 0), which is the origin of the coordinate frame in which the 3D points are output. To take advantage of the organized format and apply well-known 2D techniques to the Z image, NaN pixels must be first converted. If the direction of the *z*-axis is such that all valid 3D points have negative z components (i.e., $z\left(n_{\text{valid}}\right)<0$), then the converted point cloud ${\tilde{P}}_{n}$ is defined as

(1) $${\tilde{P}}_{n}=\left({\tilde{x}}_{n},{\tilde{y}}_{n},{\tilde{z}}_{n}\right)=\{\begin{array}{cc} P_{n} & \text{ for }n=n_{\text{valid}\;}\\ \left(0,0,z_{min\;}\right) & \text{ for }n=n_{NaN} \end{array}$$

where $z_{\text{min}}$ is the minimum of all valid $z\left(n_{\text{valid}}\right)$. Then, a z-cut at the level d>0 can be defined as the binary matrix $Z_{d}$ of the size $N_{\text{row}}\times N_{\text{col}}$, such that

(2) $$Z_{d}\left(row,\;col\;\right)=\{\begin{array}{c} \;\\ \; \end{array}\begin{array}{ll} 1\;if & \tilde{z}\left(row,\;col\right)\geq z_{max}-d\\ & 0\;\text{otherwise} \end{array}$$

where $z_{\text{max}}$ is the maximum of all valid $z\left(n_{\text{valid}}\right)$.

**Figure 5.**
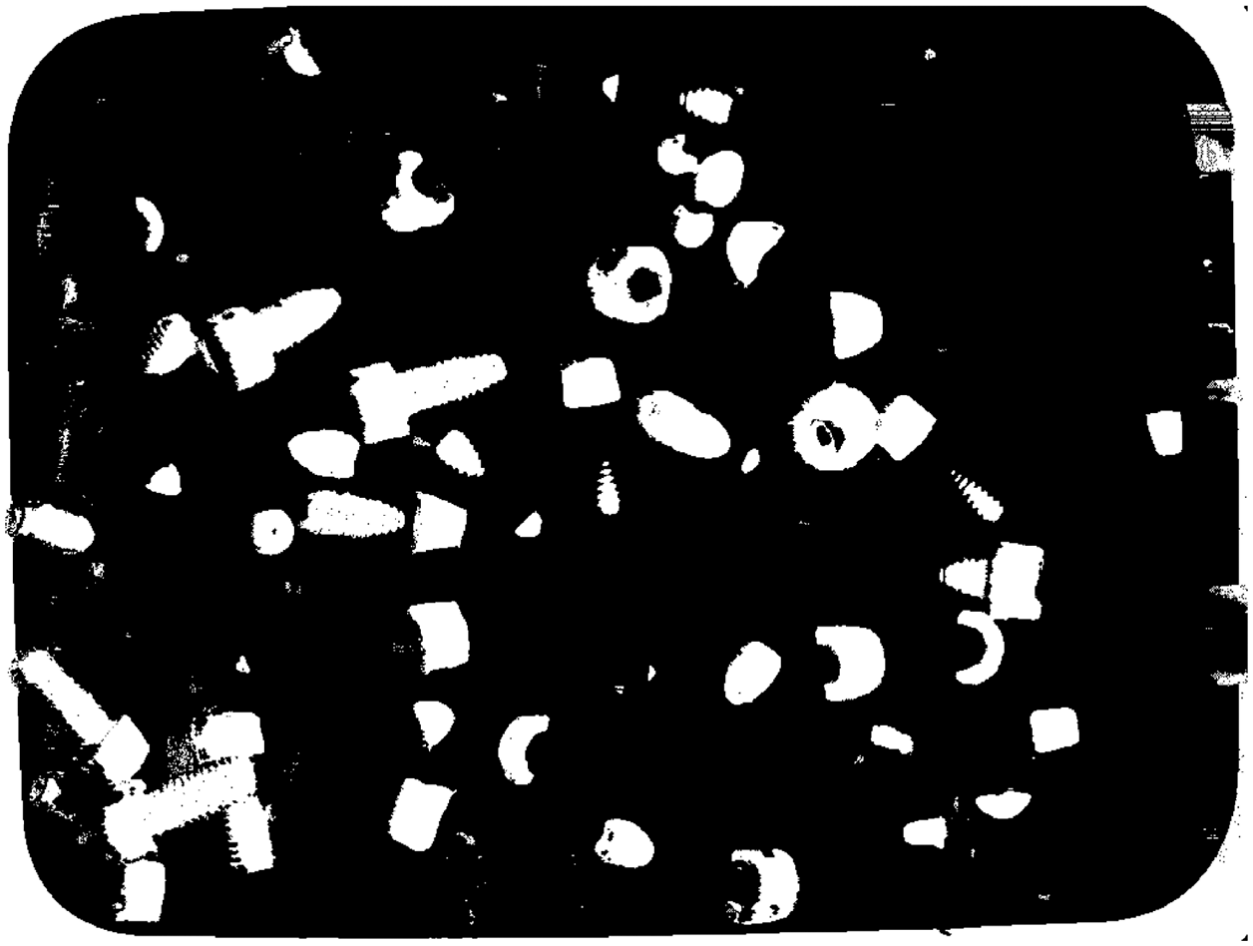
Example of z-cut and the resulting binary map $Z_{d}$ for the depth map shown in Figure 1. White pixels are 3D points with $\tilde{z}>-550\;mm$.

An example z-cut at $Z_{d}$ is shown in Figure 5. As can be seen, the binary mask consists of many disjointed clusters $C_{d}(k),k=1,\ldots ,K$, each characterized by its area $\mu_{d}(k)$, expressed as a number of connected pixels in a single cluster $C_{d}(k)$.

**Figure 6.**
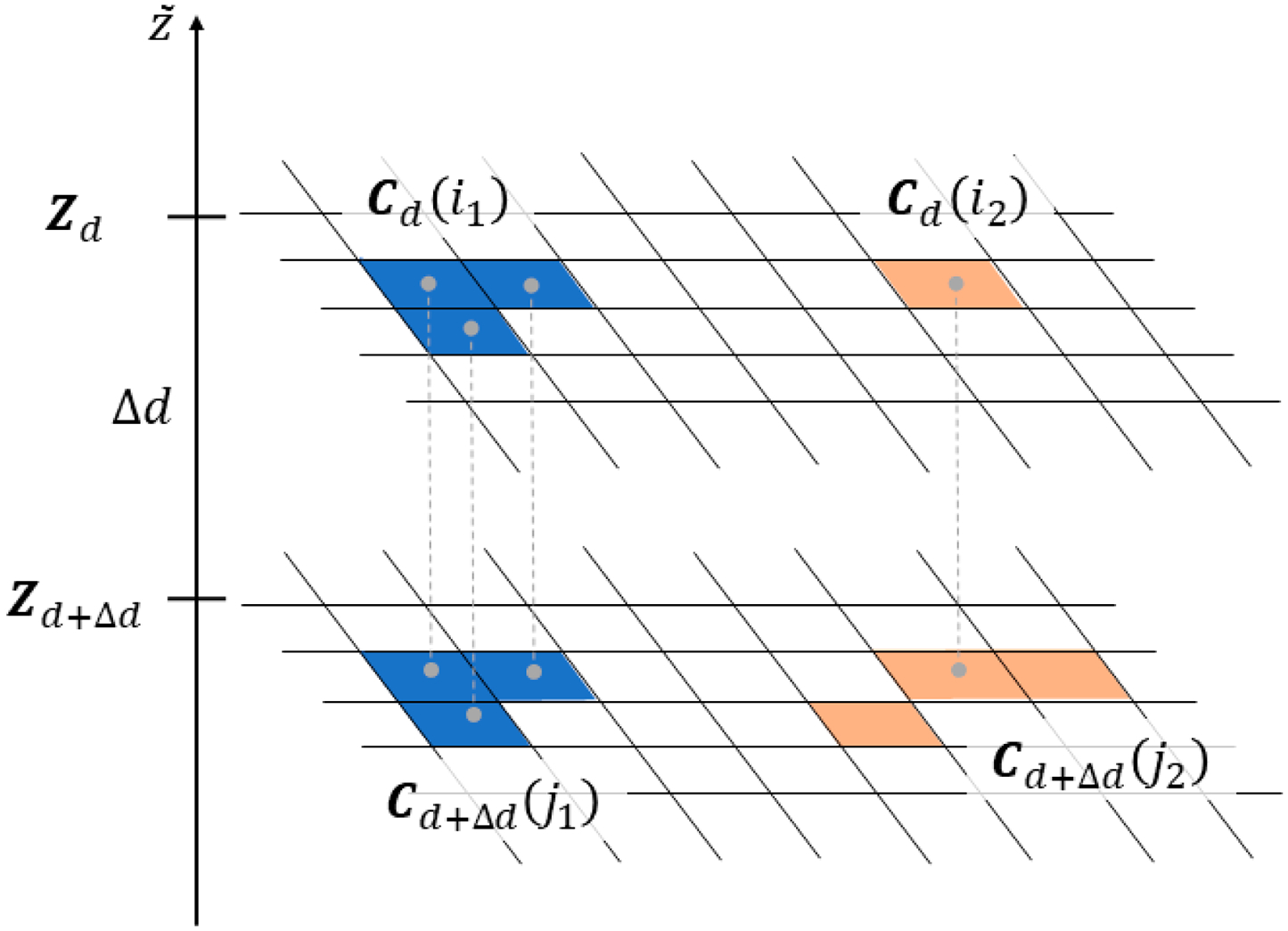
Schematic drawing illustrating three possible relations between clusters on two z-cuts: $Z_{d}$ and $Z_{d+\Delta d}$.

The proposed filtering method is based on the observations, which are illustrated in Figure 6. Any two clusters $C_{d}(i)$ and $C_{d+\Delta d}(j)$ belonging to two z-cuts $Z_{d}$ and $Z_{d+\Delta d}$ may be in one of the three relative positions on the binary maps:

1. they do not have any common points, e.g., $C_{d}\left(i_{1}\right)\cap C_{d+\Delta d}\left(j_{2}\right)=\emptyset$ as in Figure 6;
2. one is a subset of another, e.g., $C_{d}\left(i_{2}\right)\subset C_{d+\Delta d}\left(j_{2}\right)$;
3. they are the same, e.g., $C_{d}\left(i_{1}\right)\cap C_{d+\Delta d}\left(j_{1}\right)=C_{d}\left(i_{1}\right)\cup C_{d+\Delta d}\left(j_{1}\right)$, and they have the same area μ (units are number of pixels).

Each cluster $C_{d}(i)$ determines a corresponding set of 3D points $G_{d}(i)$, which is a subset of the entire organized point cloud $\tilde{P}$ (i.e., $G_{d}(i)\subset \tilde{P}$). Thus, two identical clusters $C_{d}(i)$ and $C_{d+\Delta d}(j)$ define a subset of 3D points $G_{d}(i)\equiv G_{d+\Delta d}(j)$ such that they are separated from the remaining points $\tilde{P}\setminus G_{d}(i)$ by at least Δd. These points are considered outliers since they are floating in space unconnected to the remaining data points acquired from surfaces of real, physical objects (which are in mechanical equilibrium, supported by the bottom of the bin). Finding two identical clusters on two z-cuts $Z_{d}$ and $Z_{d+\Delta d}$ is expected to be rare for datasets of reasonable quality as the majority of clusters are in the configuration 2, i.e., one is a subset of another, see clusters $C_{d}\left(i_{2}\right)$ and $C_{d+\Delta d}\left(j_{2}\right)$ in Figure 6. Note that z-cuts can be constructed only from the organized point cloud and the proposed method is applicable only to datasets in this format.

**Figure 7.**
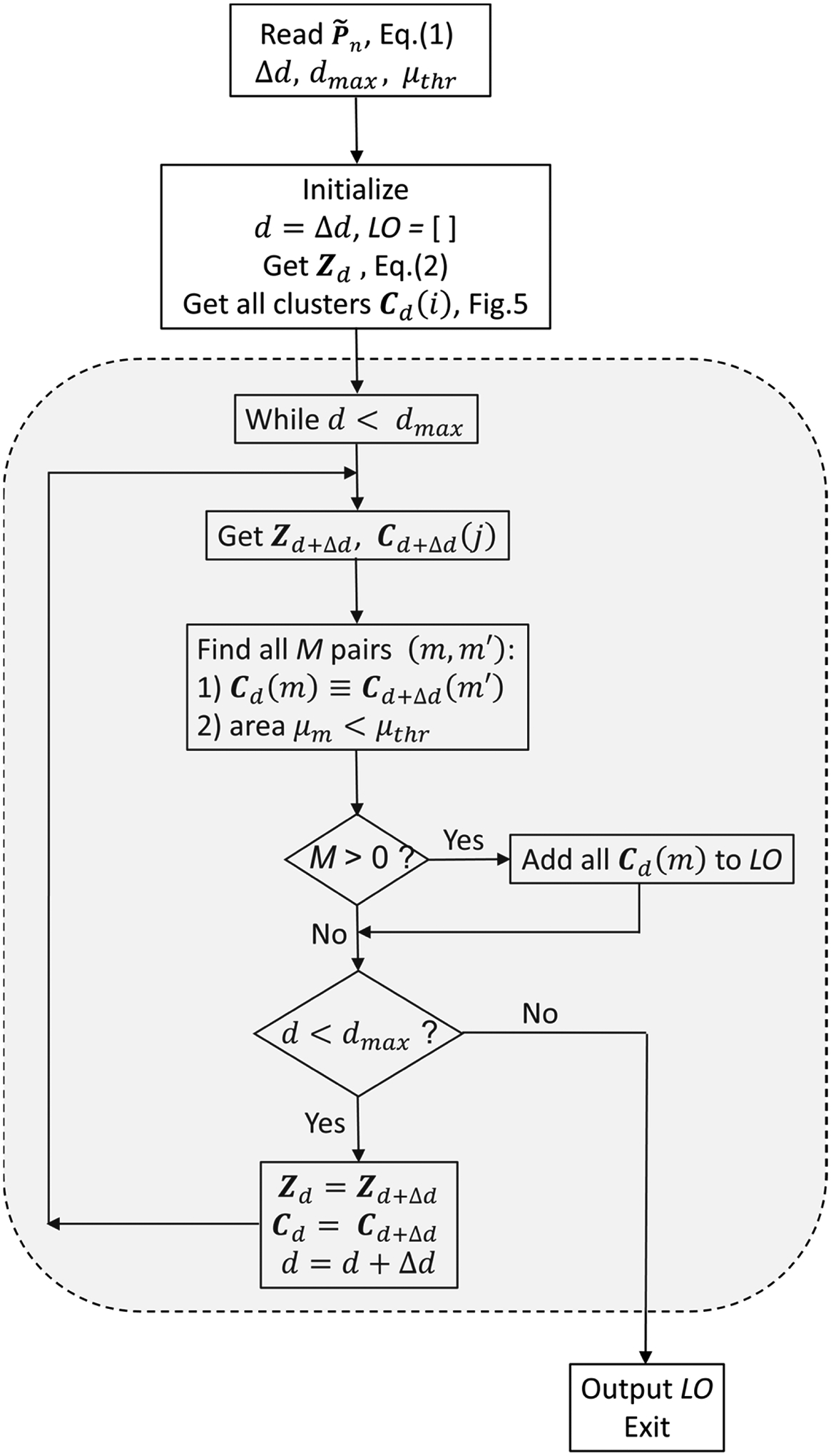
Flowchart of the proposed procedure.

In Figure 7, a schematic flowchart of the new procedure is shown. In a nutshell, the new procedure tags the outliers by running a loop indexed by depth d from $d_{min}=\Delta d$ to $d_{max}$ with step Δd. For each depth d, it checks two binary matrices, $Z_{d}$ and $Z_{d+\Delta d}$ defined in (2) and searches for pairs of identical clusters $C_{d}(m)\equiv C_{d+\Delta d}\left(m^{'}\right)$ which have areas smaller than a predefined threshold, i.e., $\mu_{d}(m)=\mu_{d+\Delta d}\left(m^{'}\right)<\mu_{thr}$. If any such M pairs are found, then the indices corresponding to the pixels in the identified clusters define a subset of 3D points, which are separated from all other data points by at least Δd distance. As explained earlier, such floating points cannot be acquired from real, physical objects and are therefore labeled as outliers in the organized 3D point cloud and are appended to the list of outliers LO.

As mentioned earlier in Section 2, generic SOR algorithms filter out outliers by thresholding a statistical parameter calculated for each datapoint, e.g., the mean or standard deviation of distances to the nearest points. The distances calculated for points in the clusters identified by the proposed method can be quite small, and then, SOR-type procedures would misclassify them as inliers. Filtering adopted by the proposed method, as explained in Figure 6, is based on a different principle and is not vulnerable to the pitfalls of thresholding. This difference causes efficient filtering by the proposed procedure and SOR missing a majority of the outliers relevant for bin-picking, as demonstrated on datasets acquired in the experiment described in the next section.

## 4. Experiment

**Figure 8.**
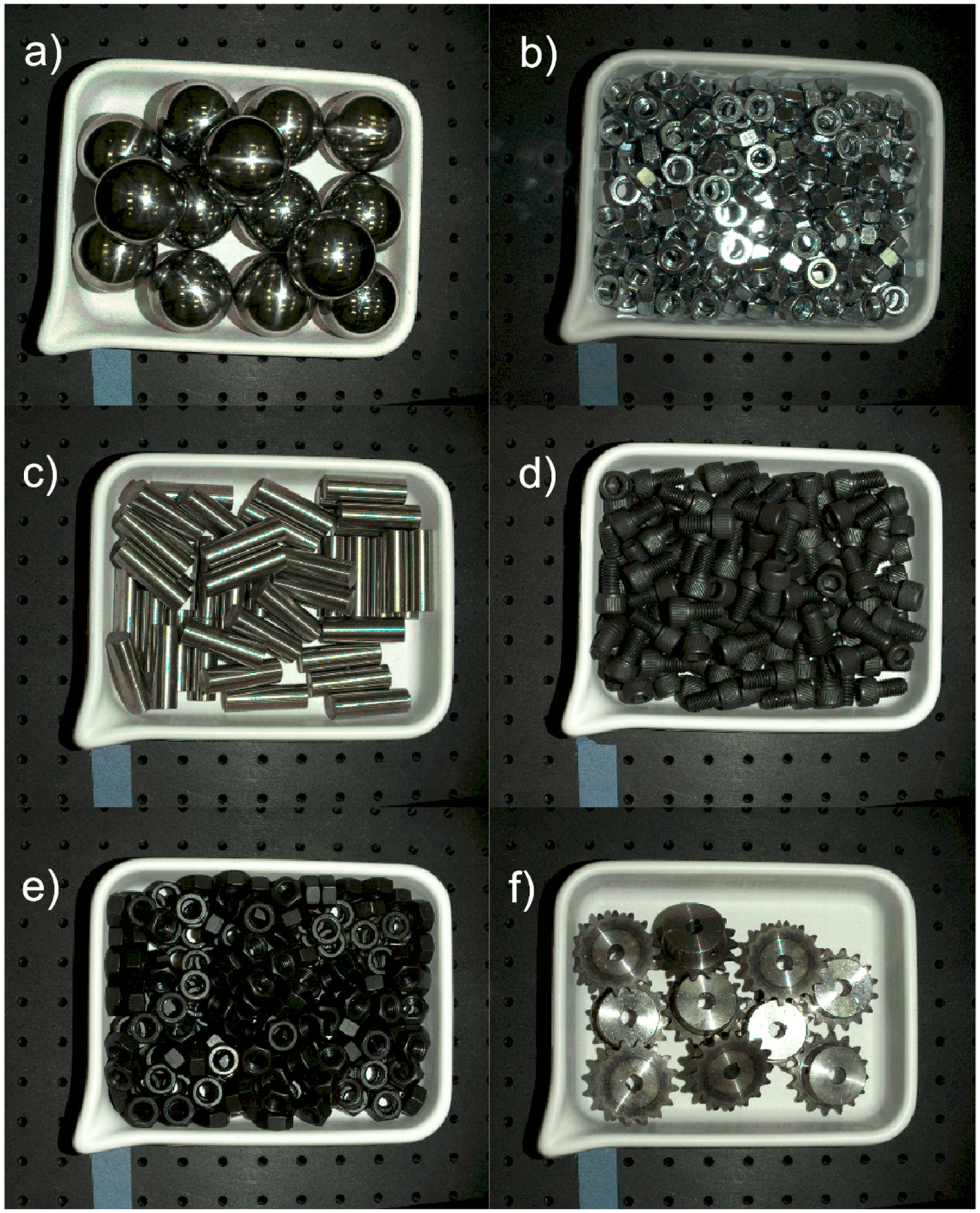
RGB images of bins filled with six different parts used in the experiments.(**a**) metal spheres; (**b**) M12 zinc nuts; (**c**) M16 metal rods; (**d**) M12 black screws; (**e**) M12 black nuts; (**f**) sprockets.

Six different types of parts were scanned with a Zivid One+ Small line-of-sight sensor (Zivid. Oslo Norway) (Disclaimer: Certain commercial equipment, instruments, or software are identified in this paper to foster understanding. Such identification does not imply recommendation or endorsement by NIST, nor does it imply that the equipment or software identified are necessarily the best available for the purpose), which is based on structured light technology that combines multiple captured images. Figure 8 shows the selection of parts with different geometries, materials, and textures (ranging from black oxide to very shiny alloy steel) that were used in this study.

A plastic bin was filled with the parts and placed on a table. Sensor was rigidly mounted above the table so the bin was in the sensor’s field-of-view (FOV) and the approximate distance between the sensor and the bin center was 0.5 m. The application programming interface (API) of the sensor provided different custom settings available to the user. Data for non-shiny parts were acquired with default settings, while shiny parts were scanned with a few different settings designed specifically for these types of objects. No extra effort was made to find the best possible combination of acquisition parameters that could yield the best 3D point clouds (i.e., with the least number of outliers) as the aim of this study was to demonstrate the performance of the proposed filtering algorithm. Since data post-processing was focused on filtering and no further action was performed (like segmentation of individual parts in a bin and calculation of their 6DoF poses), robot was not used in these experiments. Each bin filled with one kind of parts was scanned once and for each scan, two files were saved: a color RGB image file and a point cloud in an organized PLY format [36], respectively. In addition, the empty bin was scanned in five different locations on a table within the instrument’s FOV. Multiple scanning positions were needed to acquire data covering the entire surface of the bin.

## 5. Data Processing

All data post-processing was done on a desktop computer with an Intel Xenon E-2186G 3.8 GHz processor and 64 GM RAM (Intel Corporation, Santa Clara, CA, USA), using Matlab R2021a and its Image Processing Toolbox. The major steps of the processing pipeline are described in the following subsections.

### 5.1. Building Bin Model

**Figure 9.**
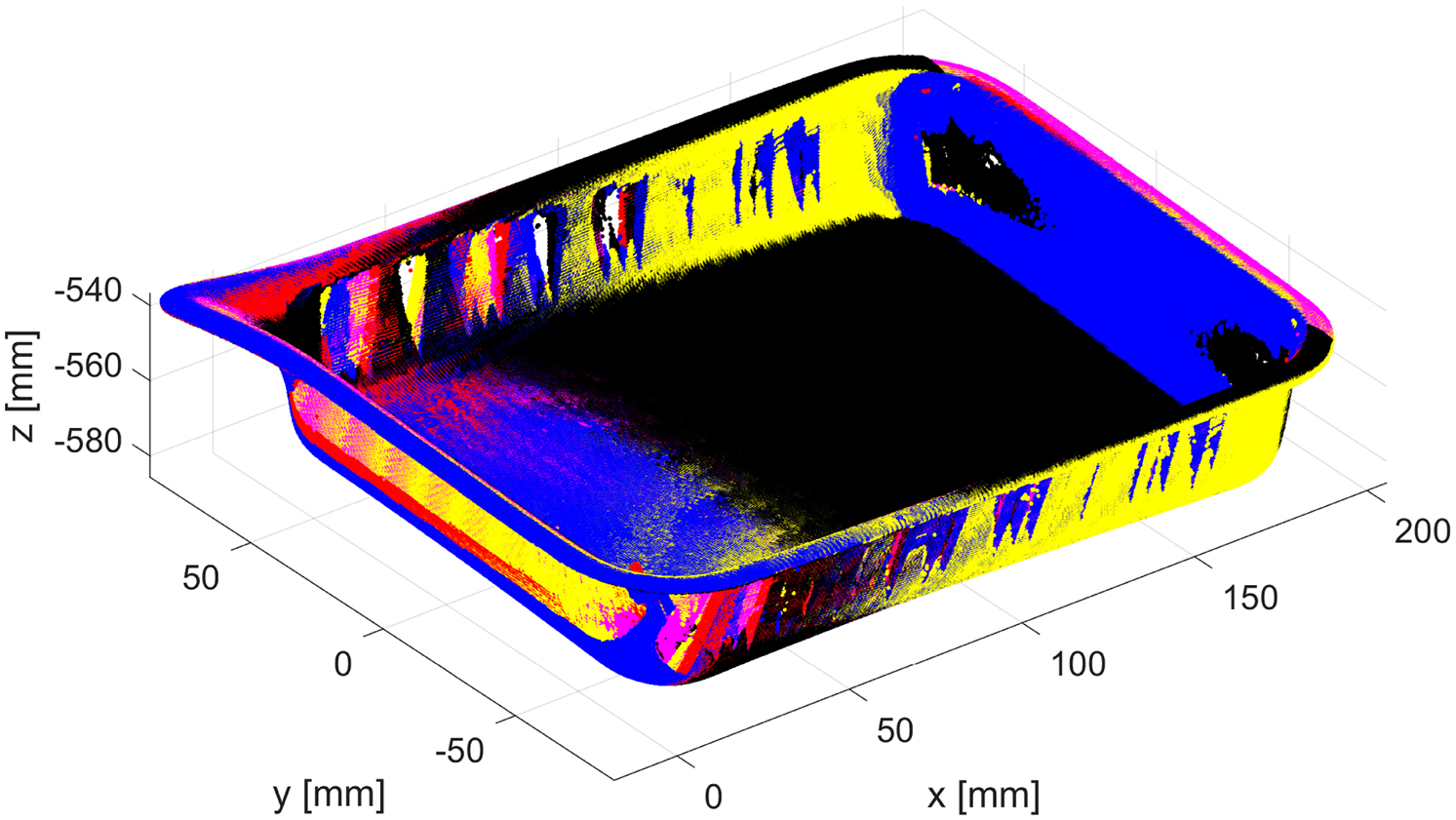
Five registered 3D point clouds representing the model of an empty bin. The different colors correspond to the individual point clouds.

First, the empty bin data is processed. The RGB image is converted to a grayscale image using the *rgb2gray()* function. The binary mask of a bin is obtained using threshold segmentation applied to the grayscale image, followed by the image dilation function, *imdilate(),* and the identification of disjoint clusters function, *bwconncomp()*. Once the binary mask of a bin is obtained, a subset of 3D points is formed from the corresponding pixels with valid entries in the PLY file. The same steps are repeated for every empty bin dataset and the resulting segmented subsets are registered to the common coordinate frame using *pcregistericp()*. The size of the resulting set is reduced using *pcdownsample(),* and this set serves as a model of the bin, as shown in Figure 9. Since the same bin is reused for all six types of parts, this step is executed only once. If a CAD model of a bin is available, this entire step can be skipped.

### 5.2. Removing Table and Bin Datapoints

Next, each of the six datasets containing a bin filled with a given type of part is processed. First, the top part of the bin is segmented from the RGB image (such as the ones shown in Figure 8) using steps similar to those described above for the empty bin data.

**Figure 10.**
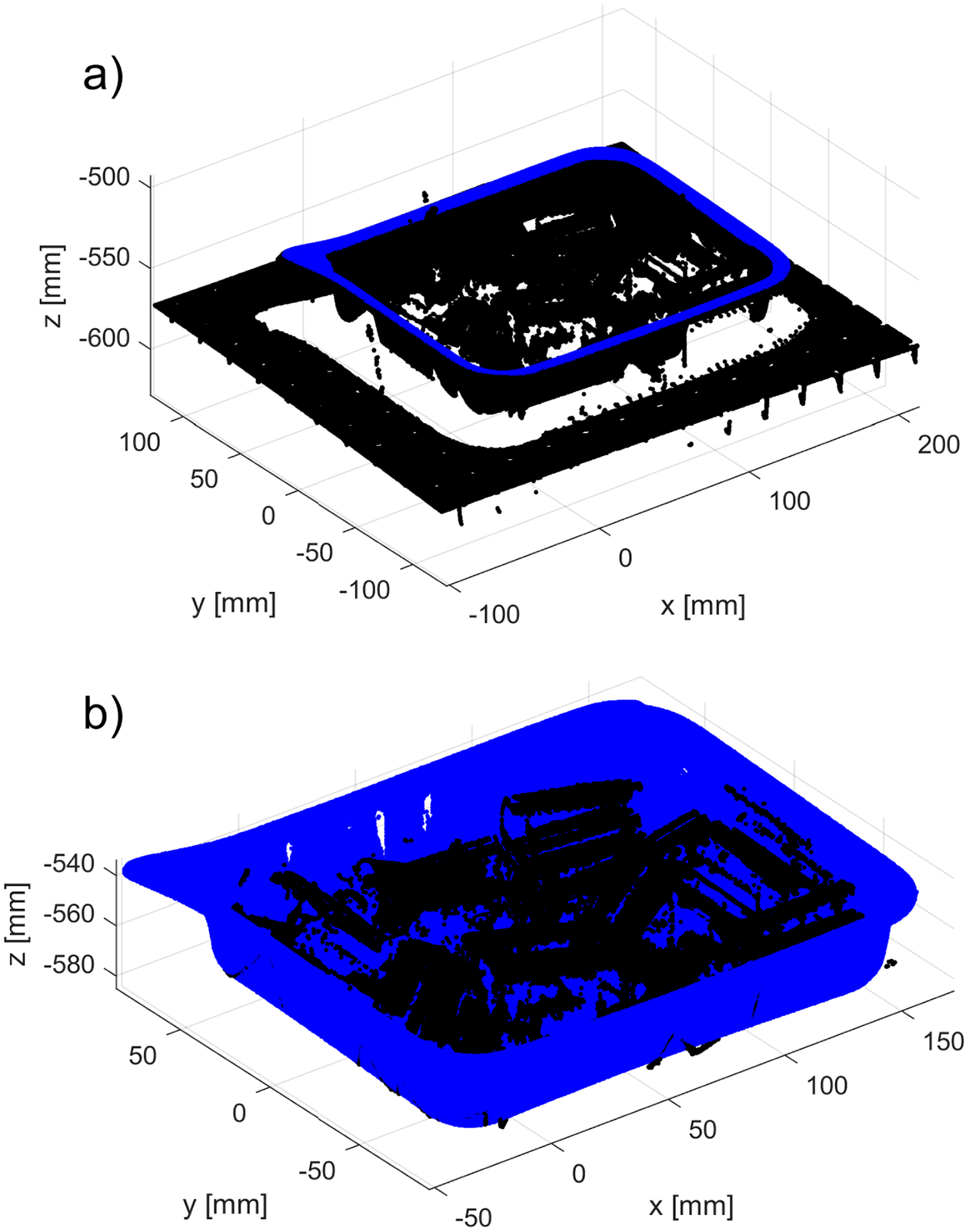
Example of removal of the points belonging to a table and the bin: (**a**) original dataset with segmented top of the bin (marked in blue)—all points on XY plane outside of the segmented top of the bin are labeled as belonging to a table; (**b**) segmented parts in the bin (marked in black) and registered model of the bin (marked in blue).

An example of the resulting 3D point cloud is shown in blue in Figure 10a. All pixels outside of the corresponding binary map are labeled (i.e., part of the data acquired from the table). The model of the empty bin is then registered to the top part of the filled bin (colored in blue in Figure 10a) using *pcregistericp(),* and the result is shown in Figure 10b.

All 3D points that are located above the top part of the bin (plus some z offset) are labeled (these were easy-to-identify outliers, such as those marked in red in Figure 3a). All remaining 3D points (marked as black in Figure 10b) that are closer than a predefined distance δ=0.1mm to any point belonging to the registered empty bin (marked as blue in Figure 10b) are also labeled. The remaining 3D points form a set to which the core outlier removal procedure is applied. To maintain the organized format of the original PLY file, all labeled 3D points are kept, but their z coordinate is set to the corresponding z coordinate of the bottom of the registered bin.

### 5.3. Gauging Performance of Both Filtering Methods

To compare the proposed filtering method with the standard SOR procedure, the organized point cloud resulting from the steps described earlier is first processed with *pcdenoise()* (Matlab implementation of SOR). With default settings, this procedure calculates for each datapoint a mean distance to the four nearest neighbors and then the standard deviation γ of all mean distances. Outliers are labeled by selecting all datapoints with a mean distance larger than γ. Only a top part of the point cloud, relevant in bin-picking applications, is processed. Then, the proposed filtering is applied to the same top portion of the 3D data. The organized format of the point cloud is preserved by changing the z components of the labeled 3D points, as described in the previous subsection.

The outcomes of both procedures applied to six different datasets are visually inspected for the existence of false negatives (i.e., outliers clearly mislabeled as inliers) and false positives (i.e., inliers incorrectly identified as outliers). Since no ground truth for outliers is available, the absolute filtering accuracy for each method (defined as a ratio of the number of filtered points to the number of true outliers) could not be calculated. To provide a quantitative comparison of both filtering procedures, only relative metrics can be determined. They are based on the three parameters: (1) the number of outliers $N_{\text{both}}$ identified by both procedures; (2) the number of outliers $N_{1}$ identified by SOR but missed by the new procedure; and (3) the number of outliers $N_{2}$ identified by the new procedure but missed by SOR. Based on the three numbers, the following metrics are determined:

(3) $$\alpha =N_{1}/N_{2},$$

(4) $$\beta_{n}=N_{\text{both}}/N_{n},n=1,2.$$

The meaning of α is as follows: if $N_{2}>0$ and α=0, then $N_{1}=0$ and all outliers identified by SOR are also identified by the new procedure. When α is small but nonzero, only a small fraction of all outliers found by SOR are not detected by the new procedure. Larger values of α signal an increasing number of outliers identified by SOR only and missed by the new procedure. Therefore, a small α is a sign of better performance of the new method when compared with SOR. The interpretation of $\beta_{n}$ is as follows: its small value indicates that for the *n*-th method (SOR or the new one), only a small portion of detected outliers is found by both methods, i.e., a large fraction of outliers is found exclusively by the *n*-th method. Thus, a smaller $\beta_{n}$ corresponds to a better performance of the *n*-th method.

### 5.4. Strategy to Set Parameters for New Filtering

The proposed procedure uses three input parameters: $\Delta d,d_{\text{max}}$, and $\mu_{thr}$. Their particular values can be derived from the CAD model of the part that fills a bin, the dimensions of the bin, and the characteristics of the sensor used for data collection. The first parameter (step Δd) defines two consecutive z-cuts, $Z_{d}$ and $Z_{d+\Delta d}$, which are checked inside a loop over d, as shown in the flowchart in Figure 7. If Δd is very small (Δd→0), then every point in the 3D point cloud could form a one-pixel cluster, tagged as an outlier. If Δd is very large (say, equal to the height of the bin $b_{h}$), then none of the points will be labeled as outliers. The second input parameter $d_{max}$ defines the threshold $z_{thr}=z_{max}-d_{max}$, which is the lowest z component for which a z-cut is constructed. If $d_{max}$ is too small (i.e., $z_{thr}$ is too large), then the proposed procedure filters only outliers with $z>z_{thr}$, leaving many outliers in the top zone of the pile of parts unfiltered. This is bad because many potentially good candidates for gripping reside in this zone. On the opposite end, if $d_{max}$ is too large, then the loop over depth d (from $d_{min}$ to $d_{max}$) becomes unnecessarily long and stretches the execution time. The suggested range for selecting a Δd is $[0.01,\;0.04]\times p_{min}$, where $p_{min}$ is the length of the smallest edge of a bounding box containing a part’s CAD model. Similarly, the suggested $d_{max}$ should be in the range $\left[p_{min},\;0.7b_{h}\right]$. Both recommendations assume that sensor characteristics are appropriate for acquiring data from a given part, i.e., $\sigma \ll p_{\text{min}}$, where σ is a level of sensor noise (e.g., residual error of fitting a planar target to 3D data). For the bin and parts used for this study, the selected nominal values were $\Delta d^{*}=0.2\;mm$ and $d_{\text{max}}^{*}=30\;mm$.

**Figure 11.**
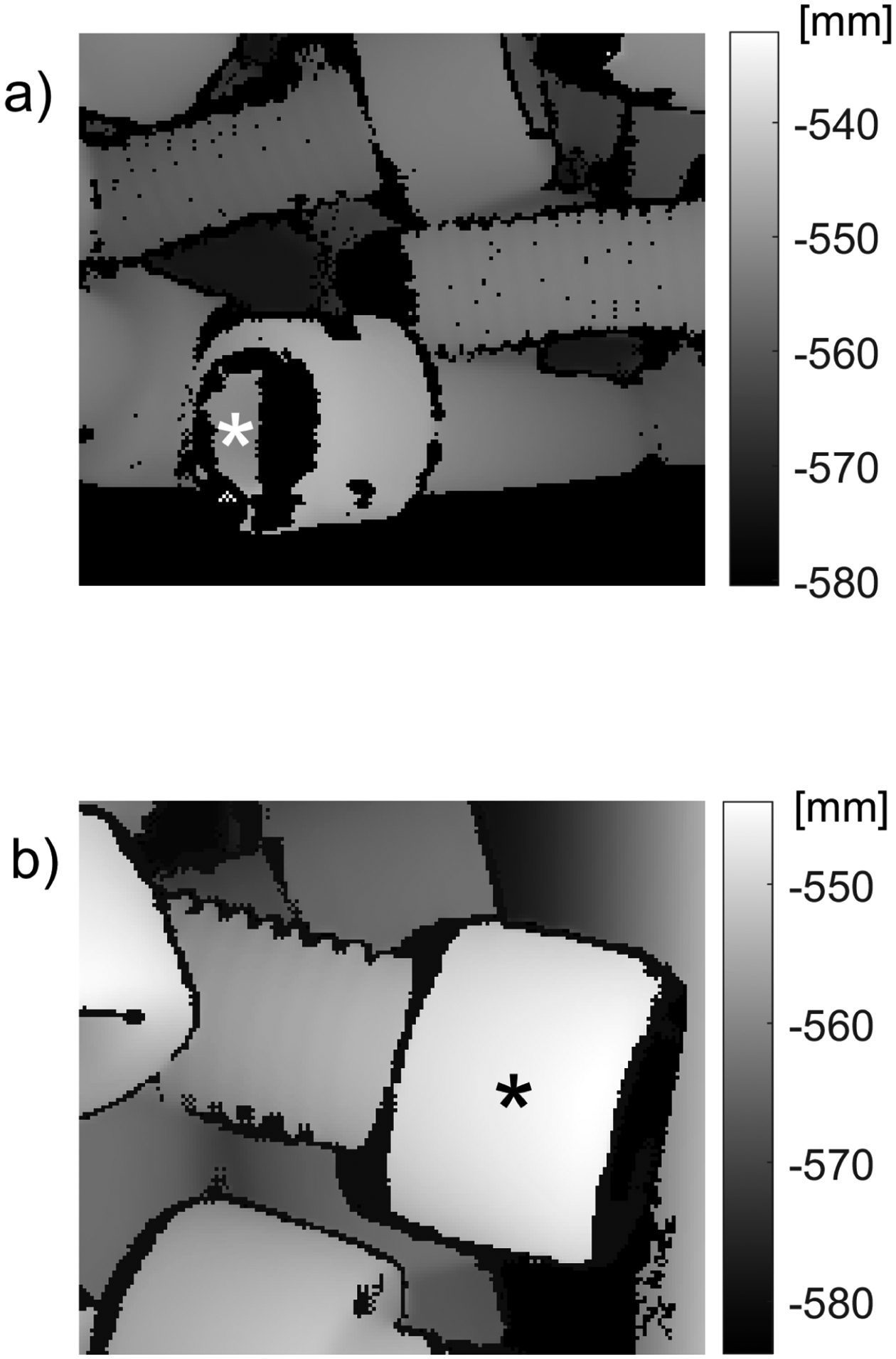
Regions of depth images illustrating the origin of incorrect outlier identification. Clusters marked by white (in (**a**)) and black (in (**b**)) asterisk are surrounded by pixels with low z (previously NaN) and the 3D points corresponding to the clusters are separated from the remaining 3D datapoints by a distance larger than Δd.

The third parameter $\mu_{thr}$ in the proposed filtering is needed to prevent the erroneous tagging of correct data points as outliers. These false positives may appear when a cluster $C_{d}(i)$ on the $Z_{d}$ map (part of the original organized 3D point cloud) is fully surrounded by NaN pixels. Two such clusters, marked by asterisks, are shown in Figure 11. A practical way to set $\mu_{thr}$ is to scan a single part placed in an empty bin in such a pose that the largest portion of the part’s surface is facing the sensor. Then, if $a_{max}$ denotes the number of points acquired from the surface, the third parameter $\mu_{thr}$ can be chosen in the range $[0.05,0.075]\times a_{max}$. A more convenient way of setting and using this parameter is to define the relative area $\rho_{d}(i)$ of the cluster $C_{d}(i)$

(5) $$\rho_{d}(i)=\mu_{d}(i)/a_{max}.$$

Then, the third parameter $\mu_{thr}$ can be replaced by the dimensionless threshold $\rho_{thr}=\mu_{thr}/a_{max}$. All six datasets presented in this study were processed with the nominal value $\rho_{thr}^{*}=0.07$. This threshold value eliminates the false positive in Figure 11b (which has a normalized area $\rho_{d}\approx 0.063$) but leaves a misclassified cluster in Figure 11a (which has a normalized area $\rho_{d}\approx 0.51$). A relatively small value of the selected threshold $\rho_{thr}^{*}$ helps to suppress the number of incorrectly identified clusters: only two were observed for the data acquired from a bin filled with M8 screws and none from the six datasets of parts shown in Figure 8. In addition, a small value of $\rho_{thr}$ ensures that only a small portion of 3D points acquired from a surface of a single part may be incorrectly removed as outliers.

To check how different values of the parameters $d_{max}$ and $\rho_{thr}$ impact a performance of the proposed procedure, it was run for other than nominal parameter values. In addition to (3), four other metrics are calculated for low and high values of parameters: $0.5\times \left[\rho_{\text{thr}}^{*},d_{\text{max}}^{*}\right]$ and $1.5\times \left[\rho_{\text{thr}}^{*},d_{\text{max}}^{*}\right]$, such as

(6) $$\begin{array}{l} \theta_{n,Lo}=N_{n}\left(0.5\rho_{thr\;}^{\text{*}}\right)/N_{n}\left(\rho_{thr\;}^{\text{*}}\right)\\ \theta_{n,Hi}=N_{n}\left(1.5\rho_{thr}^{\text{*}}\right)/N_{n}\left(\rho_{thr}^{\text{*}}\right) \end{array},\;n=1,2,$$

and

(7) $$\begin{array}{rr} & \omega_{n,Lo}=N_{n}\left(0.5\;d_{\text{max}}^{*}\right)/N_{n}\left(d_{\text{max}}^{*}\right)\\ & \omega_{n,Hi}=N_{n}\left(1.5\;d_{\text{max}}^{*}\right)/N_{n}\left(d_{\text{max}}^{*}\right) \end{array},\;n=1,2.$$

While different values of the parameters in the new procedure do not affect a performance of SOR, they have an impact on the number of outliers $N_{\text{both}}$ found by both procedures and, therefore, may change both $N_{1}$ and $N_{2}$.

## 6. Results

**Figure 12.**
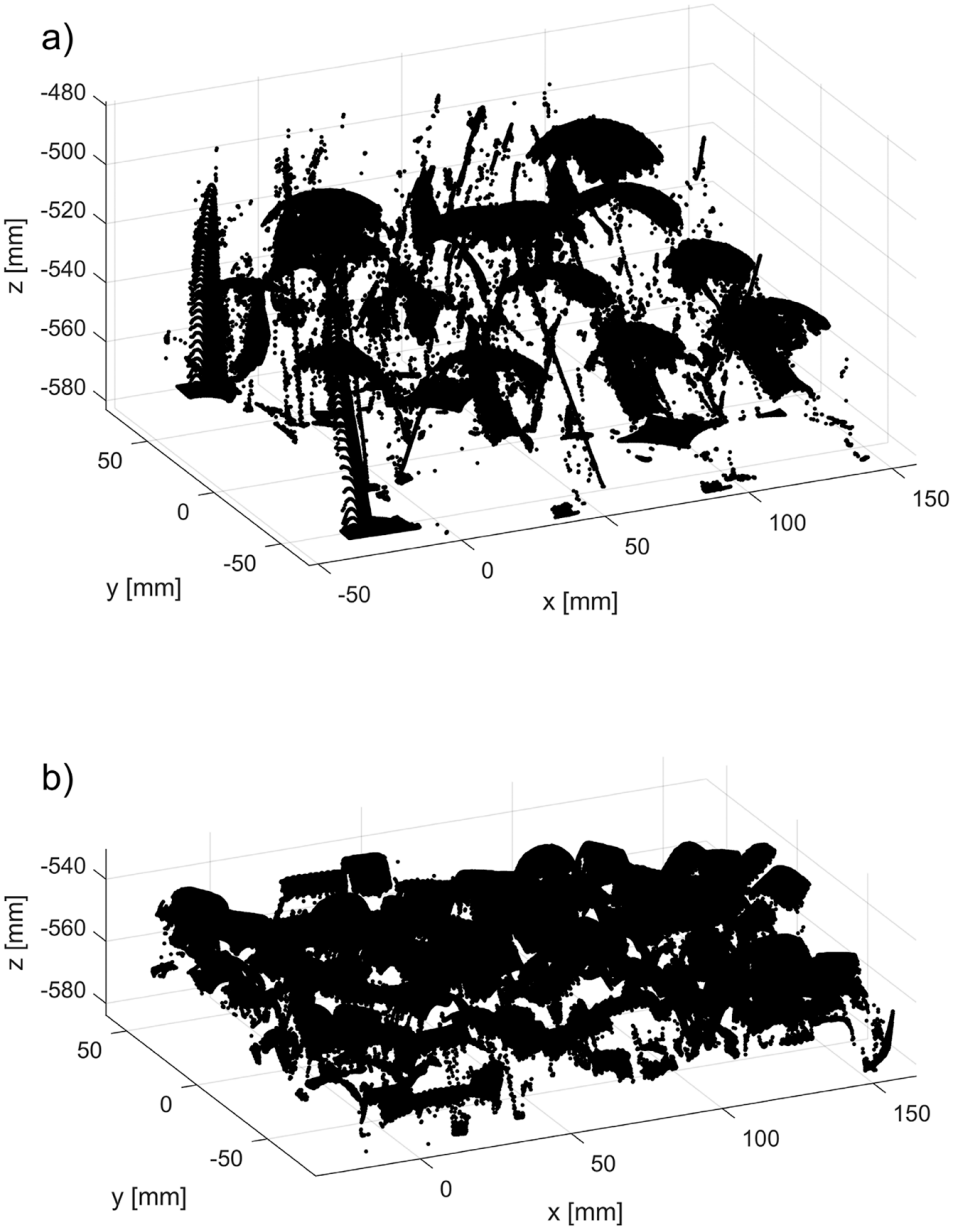
Examples of two preprocessed point clouds ${\tilde{P}}_{n}$, with table and bin points removed: (**a**) parts shown in Figure 8a; (**b**) parts shown in Figure 8d.

The outcome of the preprocessing procedure (described in Section 5.2) in which the table and the bin datapoints are removed, is shown in Figure 12. Generally, all organized 3D point clouds acquired from a bin filled with shiny parts, such as those shown in Figure 8a–c,f, contain a lot of clearly noticeable outliers, such as those shown in Figure 12a. Nonglossy parts, such as those shown in Figure 8d,e, yield relatively clean 3D point clouds with a small number of outliers, as displayed in Figure 12b.

**Figure 13.**
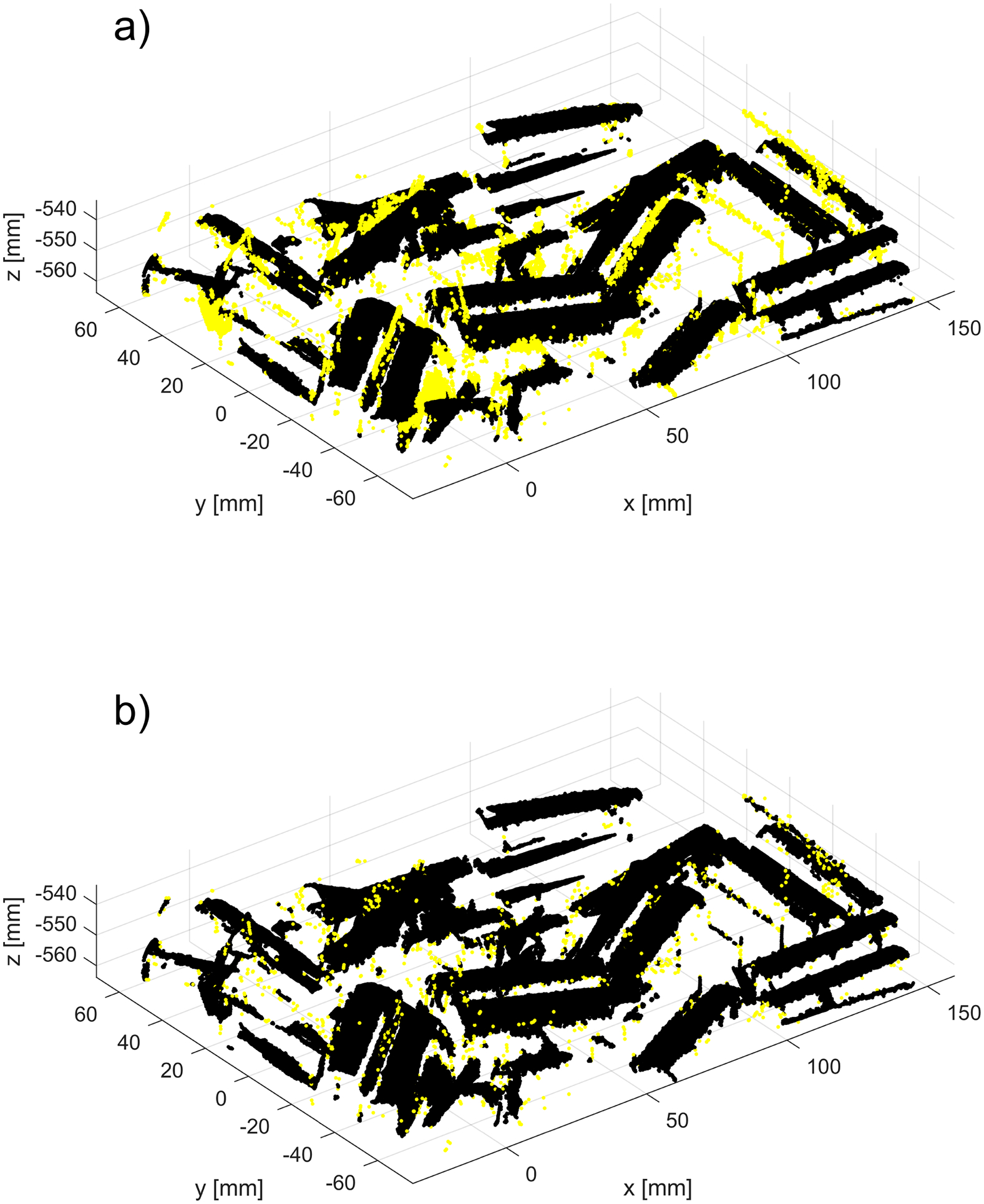
Example of filtering by (**a**) the new method run with the nominal parameters $\left(\rho_{thr}^{*},d_{\text{max}\;}^{*},\Delta d^{*}\right)$; (**b**) SOR. Outliers are plotted in yellow, the remaining part of 3D point cloud in black. RGB image of parts used in this dataset is shown in Figure 8c.

Examples of filtering the preprocessed, organized 3D point clouds ${\tilde{P}}_{n}$ by SOR and the new method run with the nominal values of parameters $\left(\rho_{thr}^{*},d_{\text{max}\;}^{*},\Delta d^{*}\right)$ are shown in Figure 13. For other shiny parts, the new method identifies much more outliers than SOR, similar to the results plotted in Figure 13.

**Figure 14.**
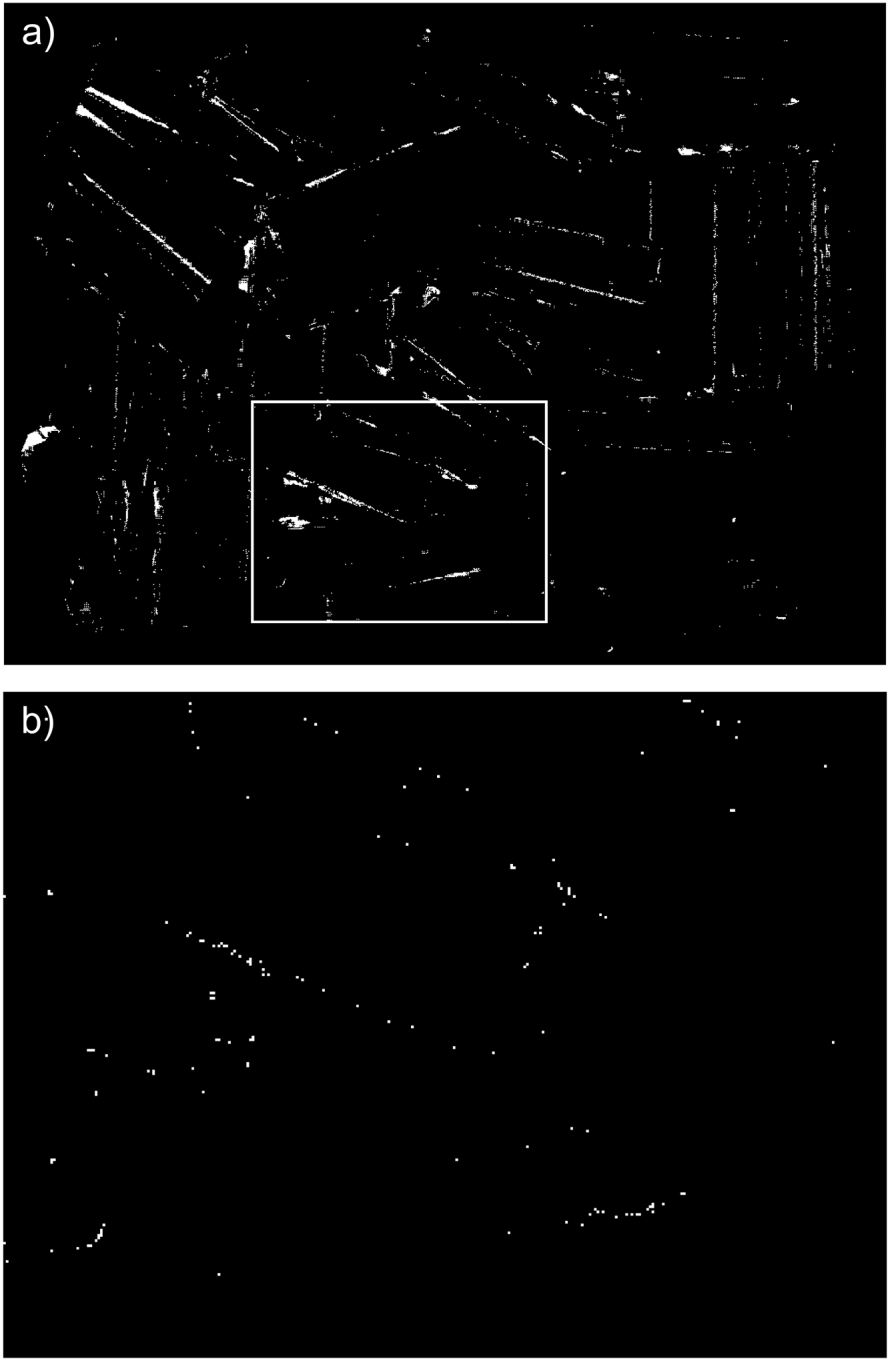
2D maps showing the locations of the outliers output by (**a**) the new method run with the nominal parameters $\left(\rho_{\text{thr}}^{*},d_{\text{max}}^{*},\Delta d^{*}\right)$; (**b**) SOR. Only a portion of the entire map generated by SOR is shown in the rectangular region defined in (**a**). Corresponding 3D plots of outliers are shown in Figure 13.

The difference between both methods can be also visualized on a binary 2D map of the same size as z-cut $Z_{d}$. In Figure 14a, white pixels indicate the locations of outliers labeled by the new procedure run with the nominal values of parameters $\left(\rho_{thr}^{*},d_{\text{max}\;}^{*},\Delta d^{*}\right)$. A rectangle marks a subregion of a full mask, which is enlarged in Figure 14b: it shows locations of outliers output by SOR that fall inside the marked subregion. The example of maps shown in Figure 14 are from the dataset containing parts shown in Figure 8c. The maps created for other shiny parts investigated in this study reveal a similar disparity between the numbers of outliers output by both methods.

**Figure 15.**
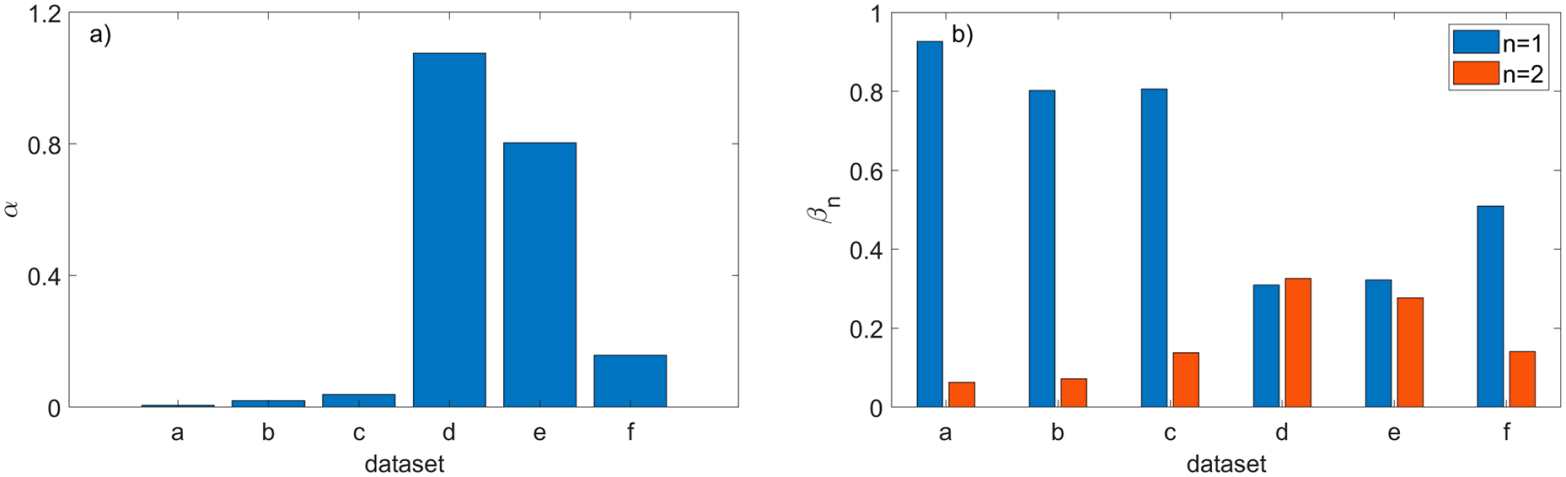
Comparison of filtering efficiency provided by SOR and the new method run with the nominal values of the parameters $\left(\rho_{thr}^{*},d_{\text{max}}^{*},\Delta d^{*}\right)$: (**a**) α ratio; (**b**) $\beta_{n}$ ratio where n = 1 is for SOR and n = 2 is for the new method.

**Figure 16.**
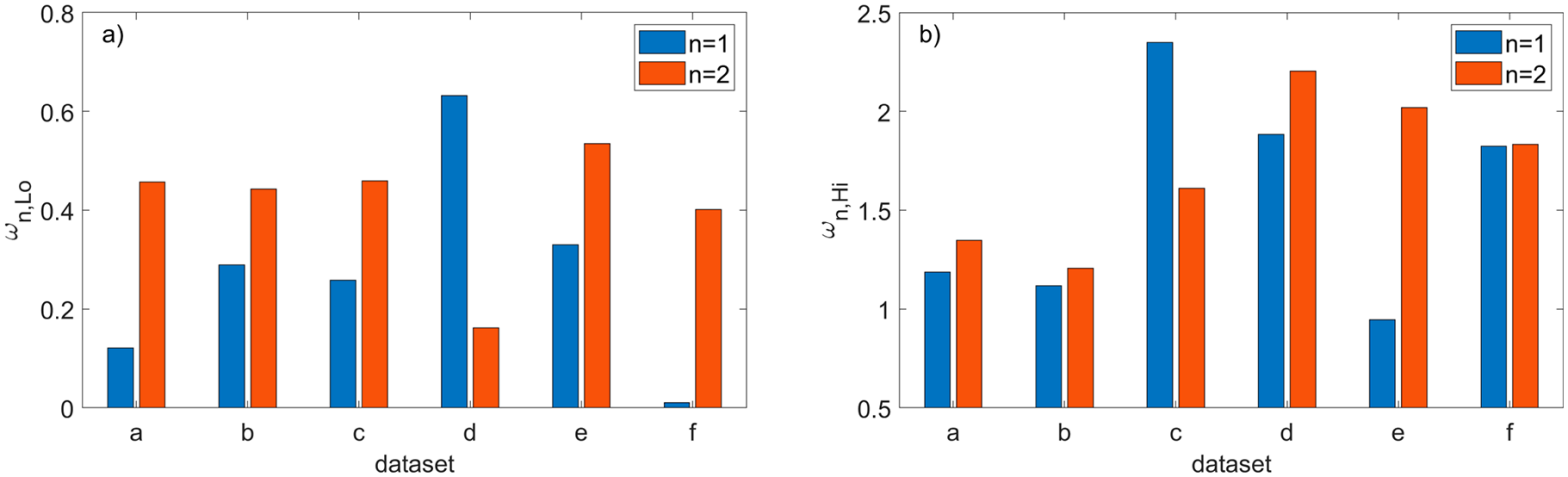
Comparison of the SOR (n = 1) with the new method (n = 2) run with the nominal values of the parameters $\left(\rho_{thr}^{*},\Delta d^{*}\right)$ and two different thresholds $d_{max}$: (**a**) $\omega_{n,Lo}$ ratio for the reduced $d_{max}=0.5d_{\text{max}}^{*}$; **(b)** $\omega_{n,Hi}$ ratio for the increased $d_{max}=1.5d_{\text{max}}^{*}$.

The quantitative metrics α and $\beta_{n}$, defined in (3), that are used to compare the filtering efficiency of both methods are shown in Figure 15. To show the consequences of running the new method with values different than the nominal values of parameter $d_{max}$ and the ratios $\omega_{n,Lo}$ and $\omega_{n,Hi}$ defined in (6) are plotted in Figure 16. The two other parameters were set to their nominal values $\left(\rho_{thr}^{*},\Delta d^{*}\right)$, and the ratio $\omega_{n,Lo}$ in Figure 16a corresponds to the reduced $d_{max}=0.5d_{max}^{*}$ while $\omega_{n,Hi}$ in Figure 16b corresponds to the larger $d_{max}=1.5d_{\text{max}}^{*}$.

**Figure 17.**
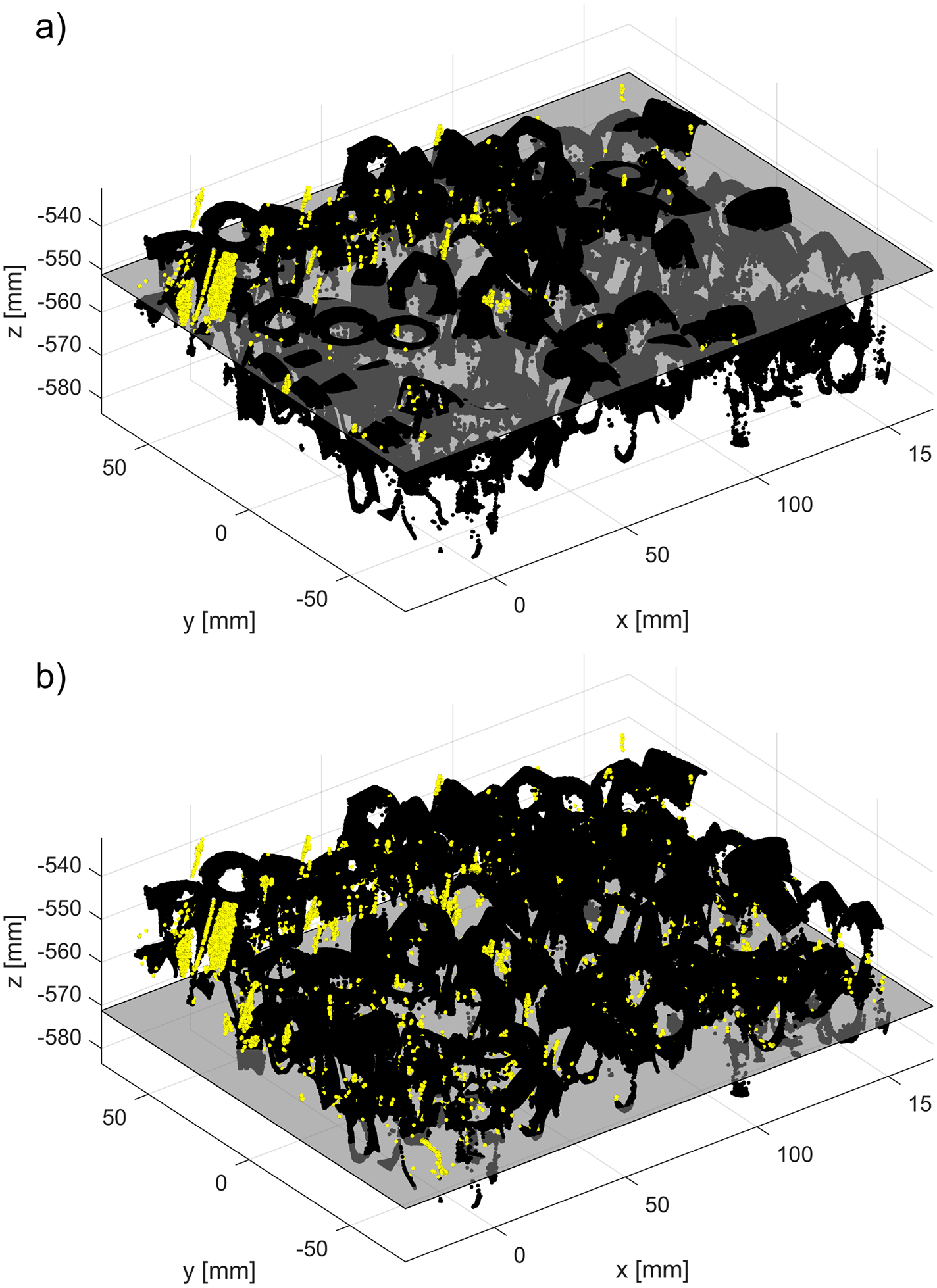
Outcomes of the new method run with the nominal values $\left(\rho_{thr}^{*},\Delta d^{*}\right)$ and two different values of $d_{max}$ : (**a**) $d_{max}=0.5{\;d}_{\text{max}\;}^{*}$; (b) $d_{max}=1.5{\;d}_{\text{max}}^{*}$. Yellow points indicate identified outliers, black points are the remaining points from the dataset, and a grey colored plane is added at $z=z_{thr}$. For better visualization, a scale on the z-axis is stretched by 1.5.

The example 3D plots resulting from running the new method with non-default values of the parameter $d_{max}$ are displayed in Figure 17 for the nonglossy parts shown in Figure 8e. Yellow points mark identified outliers, black points are the remaining datapoints. A plane drawn in grey is added at $z=z_{thr}$, where $z_{thr}=z_{max}-d_{max}$ and $z_{max}$ is the coordinate of the highest point in ${\tilde{P}}_{n}$, as in (2). Thus, the location of the plane corresponds to the last z-cut $Z_{d}$ processed in a loop by the new procedure, as explained in the flowchart in Figure 7. Results obtained for other parts displayed in Figure 8 reveal a similar pattern, i.e., an increasing number of the identified outliers with the increasing $d_{max}$.

**Figure 18.**
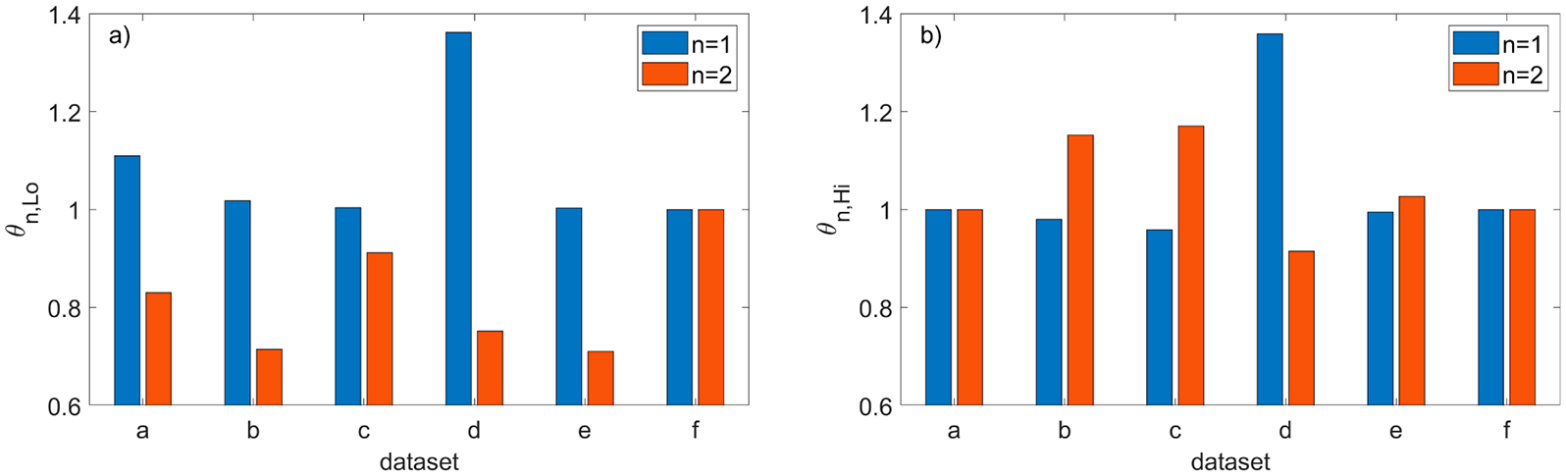
Comparison of the SOR (n = 1) with the new method (n = 2) run with the nominal values of parameters $\left(d_{\text{max}}^{*},\Delta d^{*}\right)$ and two different thresholds $\rho_{thr}$ : (**a**) $\theta_{n,Lo}$ ratio for the reduced $\rho_{thr}=0.5\;\rho_{thr}^{*};$ (**b**) $\theta_{n,Hi}$ ratio for the increased $\rho_{thr}=1.5{\;\rho }_{thr}^{*}$.

To show the consequences of running the new method with different values than the nominal values of the parameter $\rho_{thr}$, the ratios $\theta_{n,Lo}$ and $\theta_{n,Hi}$ defined in (5) are plotted in Figure 18. The two other parameters were set to their nominal values $\left(d_{max}^{*},\Delta d^{*}\right)$ and the ratio $\theta_{n,Lo}$ in Figure 18a corresponds to the reduced $\rho_{thr}=0.5\rho_{thr}^{*}$ while $\theta_{n,Hi}$ in Figure 18b corresponds to the larger $\rho_{thr}=1.5\rho_{thr}^{*}$.

**Figure 19.**
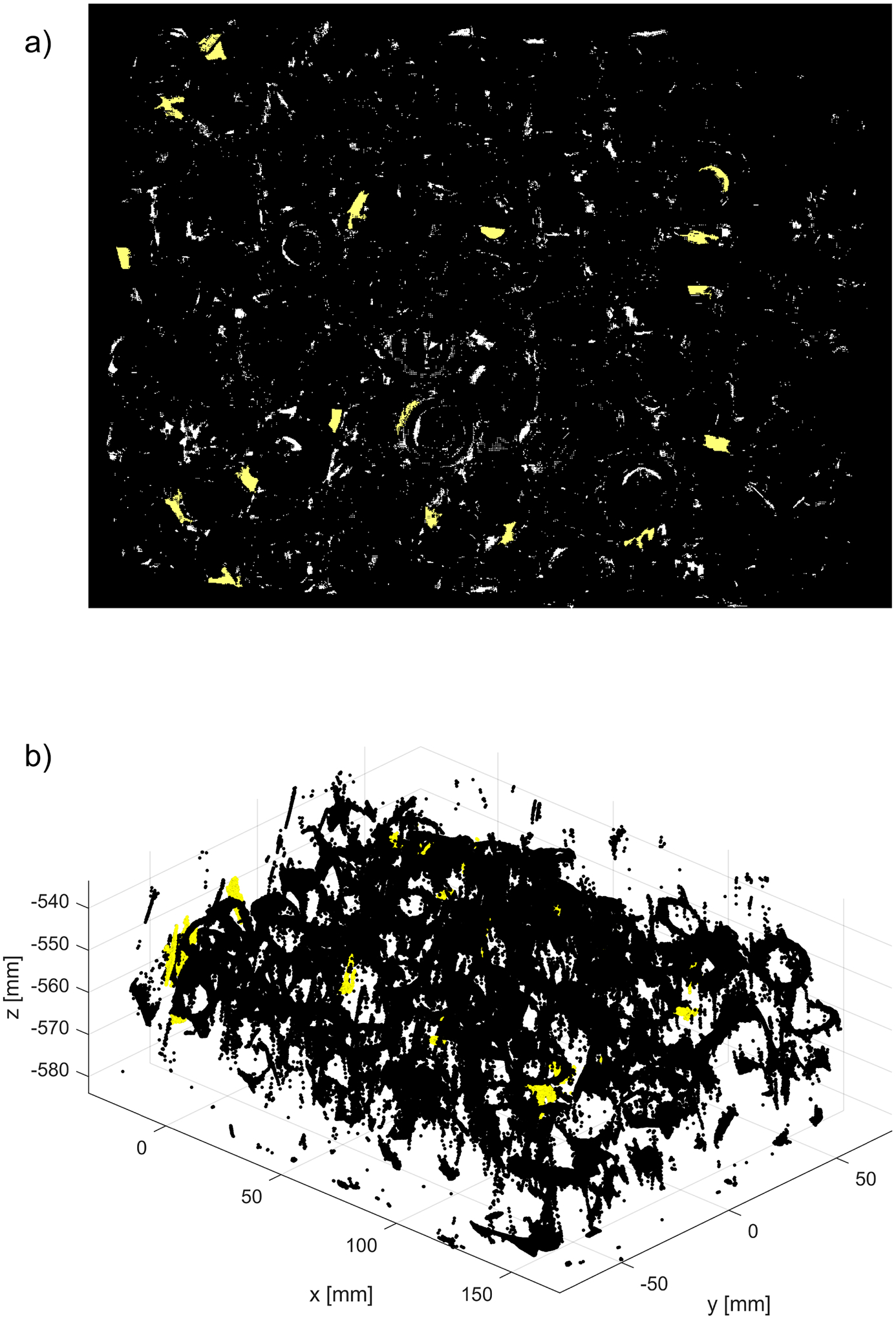
Effect of running the new algorithm with two different values of the parameter $\rho_{thr}$: nominal and reduced. In the binary map shown in (**a**), the white pixels are locations of outliers when the nominal value was used, while yellow pixels mark outliers missed when reduced, more restrictive threshold was applied. In (**b**), the whole unfiltered 3D point cloud is plotted with black points, yellow points are missed outliers displayed in (**a**).

To visualize the impact of using non-default values of $\rho_{thr}$ in the new method, a binary 2D map with the locations of outliers is shown in Figure 19a. White pixels are outliers identified by running the new method with the nominal parameter $\rho_{thr}^{*}$, while pixels displayed in yellow mark outliers missed when the smaller, more restrictive threshold $\rho_{thr}=0.5\;\rho_{thr}^{*}$ is used. In Figure 19b, the unfiltered 3D point cloud ${\tilde{P}}_{n}$ is plotted in black and the missed outliers shown in Figure 19a are plotted in yellow. The plotted results obtained from the dataset with parts are shown in Figure 8b.

**Figure 20.**
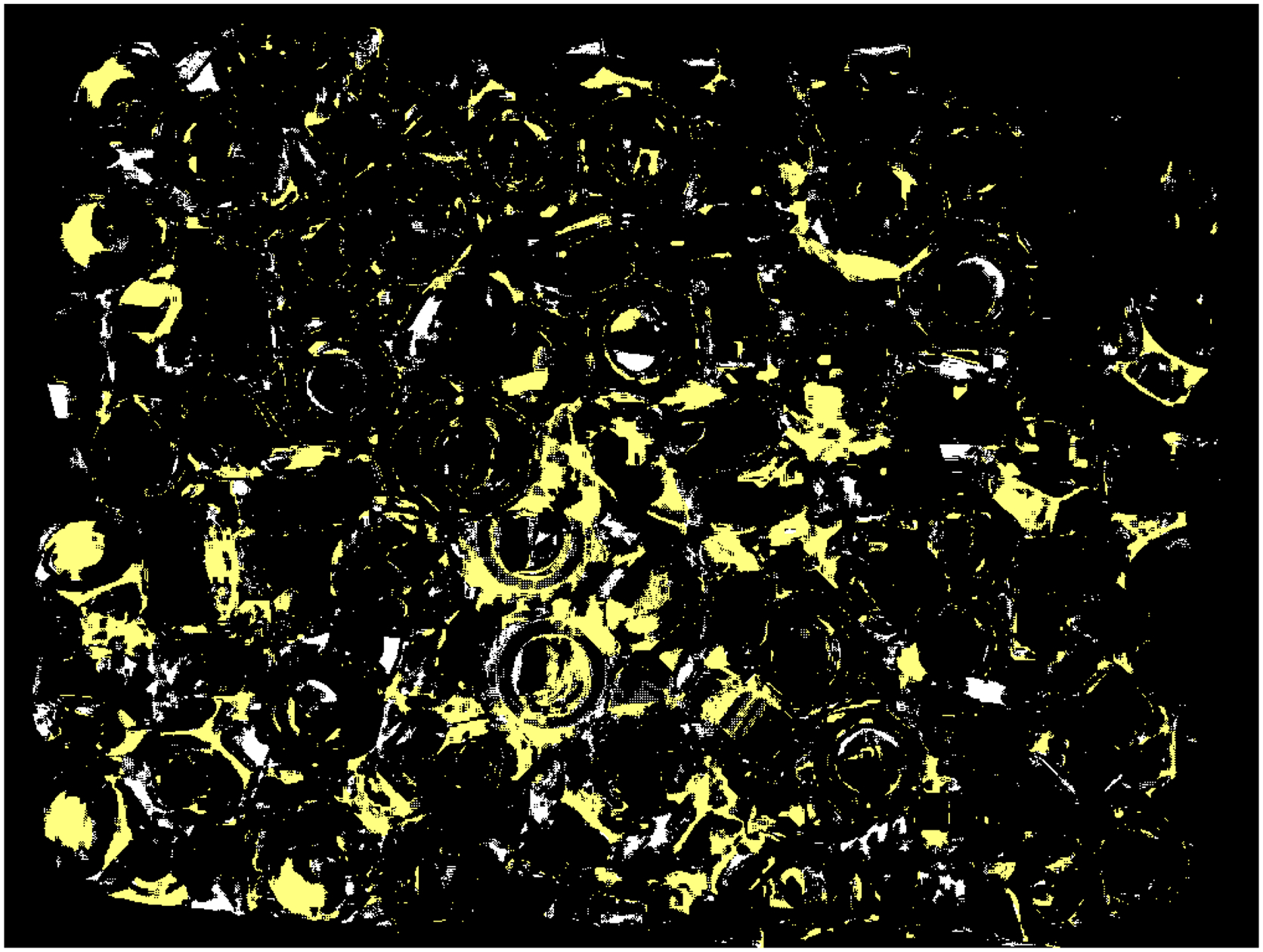
2D binary map illustrating the impact of the reduced parameter Δd on the performance of the new method. White pixels are locations of outliers when the procedure is run with the default value of Δd, yellow pixels are extra outliers identified when the reduced parameter was applied.

Finally, to visualize the impact of a reduced value of the step Δd on a performance of the new method, the 2D binary map is shown in Figure 20. The proposed method was run twice, with the default values of the parameters $\left(\rho_{\text{thr}}^{*},d_{\text{max}}^{*}\right)$ and two values of Δd: the nominal $\Delta d^{*}$ and the reduced $0.1\;\Delta d^{*}$. The white pixels are the locations of outliers found with the nominal step value, while the yellow pixels show extra identified outliers when the reduced value of the step was used. Presented in Figure 20, a map obtained for the dataset with parts is shown in Figure 8b; the maps obtained for other datasets reveal a similar pattern, i.e., an increased number of outliers for reduced step Δd. A majority of the yellow pixels are false negatives, i.e., they are 3D points which, after visual inspection, should be in the inliers category.

## 7. Discussion

The performance of the proposed filtering method, gauged by the ratios α and $\beta_{2}$, clearly outperforms the general use SOR procedure when applied to the shiny parts shown in Figure 8a–c,f. As explained in Section 5.3, both ratios play complementary roles, and their small values mean that the majority of outliers is identified exclusively by the new method. Results shown in Figure 15 confirm this conclusion for datasets (a, b, c, f). The binary 2D maps shown in Figure 14 provide graphic evidence aligned with the conclusion based on the ratios α and $\beta_{2}$. In addition, closer inspection of the 2D maps reveals a difference in the characteristics of the outliers filtered by both methods. A majority of the outliers identified by the new method are grouped in large clusters of connected pixels, as shown in Figure 14a. Contrary to this, the outliers filtered by SOR are mostly single, isolated pixels dispersed across the 2D map, as displayed in Figure 14b. That difference between both maps originates from fundamentally different filtering mechanisms: in SOR, filtering is based on the thresholding of mean local distances. Such an approach cannot identify large blobs of 3D points, as their local distances to the nearest neighbors fall below the threshold. The new method can find even large clusters on 2D maps that correspond to large groups of 3D points if their z components are separated by a distance Δd from the surrounding points in the organized point cloud.

**Figure 21.**
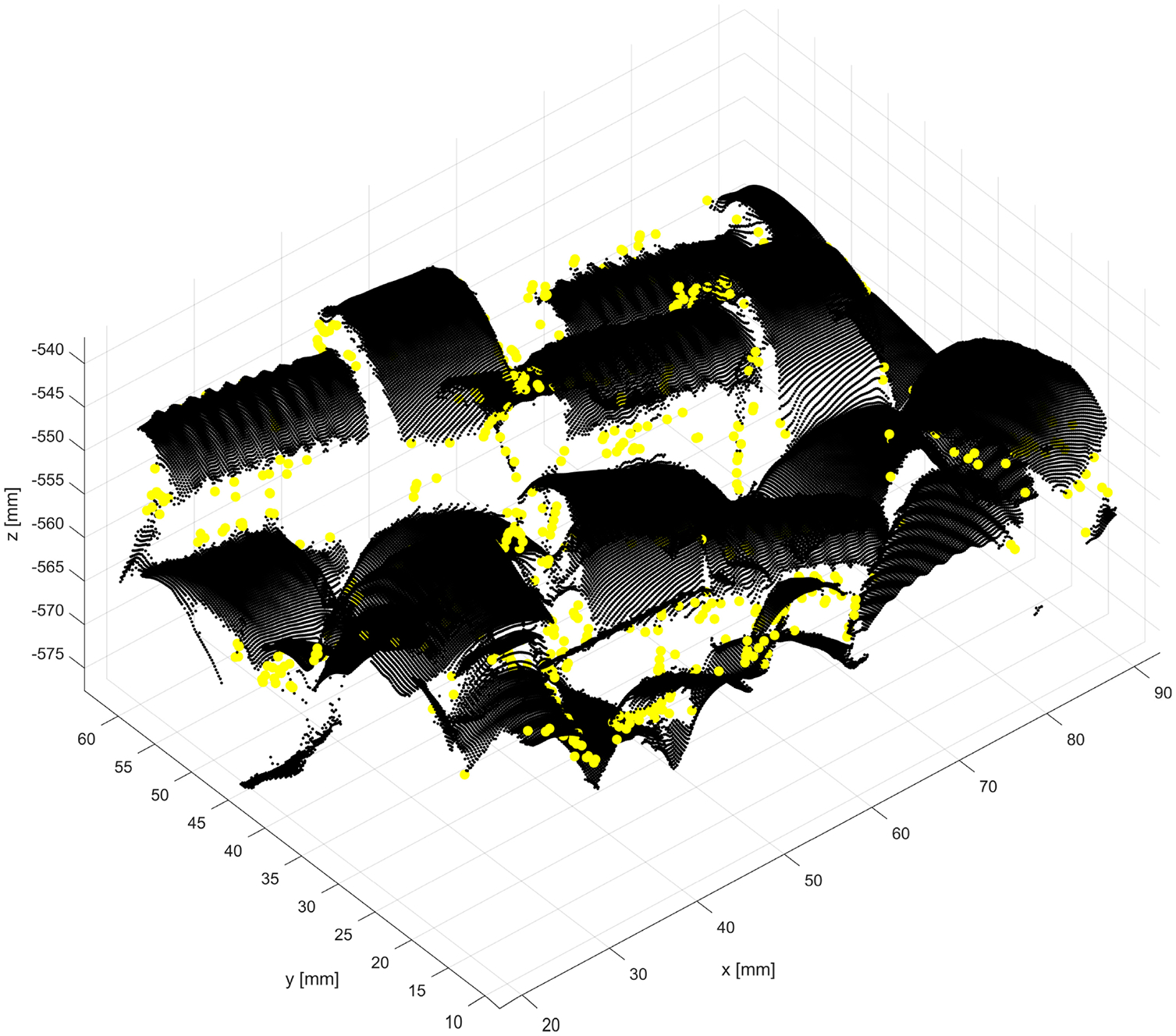
Zoomed-in portion of the organized 3D point cloud, yellow points mark outliers found by SOR, the remaining datapoints plotted as black points.

However, the ratios α and $\beta_{2}$ for nonglossy parts, such as those displayed in Figure 8d,e, clearly contradict the conclusions based on the analysis of results for shiny parts: both ratios are large for datasets (d, e), as shown in Figure 15. Relatively large values of $\beta_{2}$ cause the ratios $\beta_{1}$ to be small, which means that a substantial fraction of outliers is found only by SOR (i.e., they are missed by the new procedure). As the ground truth for outliers is not available, the observed underperformance of the new method for datasets (d, e) should be attributed to the characteristics of both datasets and properties of the ratios α and $\beta_{2}$. If the outliers identified only by SOR contain many false positives, this will incorrectly boost the total number $N_{1}$ of outliers found by SOR only and will cause a disproportional increase in the ratios α and $\beta_{2}$, as follows from (3). Compared to shiny parts, the datasets (d, e) for nonglossy parts are very clean and they contain a small number of visually detectable outliers, as demonstrated in Figure 12. This causes the standard deviation γ of mean distances to the nearest points to be small. This, in turn, affects the thresholding utilized by SOR. As a consequence, the small threshold causes the misclassification of many 3D points as outliers, in contradiction to the visual inspection. An example of such a case is shown in Figure 21. Outliers found by SOR are plotted in yellow but many of these points are located close to 3D points, correctly representing the scanned surfaces of screws. Thus, many of the yellow points are false positives, i.e., inliers misclassified as outliers.

For the same dataset (d), the new method identifies a smaller number of outliers, which agrees with the visual inspection. Thus, the overestimated number of outliers identified by SOR, paired with a smaller number of the outliers found by the new method, leads to misleading, large values of the ratios α and $\beta_{2}$, as shown in Figure 15 for datasets (d, e).

## 8. Conclusions

The two methods discussed in this paper perform the filtering of outliers based on two different principles. Both procedures find a common subset of outliers, but each of them also finds a portion of outliers that is missed by the other procedure. If the execution time is not critical, then the most outliers can be removed by applying both procedures. If time is of the essence, then the new method may be preferred, as it filters a substantial portion of outliers missed by SOR, especially in datasets acquired from shiny parts.

## Data Availability

The data presented in this study are available on request from the corresponding author.
